# Proteomic and phosphoproteomic analysis of rabies pathogenesis in the clinical canine brain and identification of a kinase inhibitor as a potential repurposed antiviral agent

**DOI:** 10.1371/journal.pone.0323931

**Published:** 2025-06-27

**Authors:** Peerut Chienwichai, Kunjimas Ketsuwan, Boonlert Lumlertdacha, Chanon Fa-Ngoen, Punchaya Supasawat, Rojjanaporn Pulmanausahakul, Promsin Masrinoul, Tipparat Thiangtrongjit, Onrapak Reamtong

**Affiliations:** 1 Princess Srisavangavadhana Faculty of Medicine, Chulabhorn Royal Academy, Bangkok, Thailand; 2 Research Center on Clinical and System Microbiology (RCSyM), Chulabhorn Royal Academy, Bangkok, Thailand; 3 Institute of Molecular Biosciences, Mahidol University, Nakhon Pathom, Thailand; 4 Queen Saovabha Memorial Institute, Thai Red Cross Society, WHO Collaborating Center for Research and Training on Rabies Prophylaxis, Bangkok, Thailand; 5 Department of Molecular Tropical Medicine and Genetics, Faculty of Tropical Medicine, Mahidol University, Bangkok, Thailand; McGill University, CANADA

## Abstract

Rabies is a fatal zoonosis caused by the rabies virus (RABV) that has afflicted humans for thousands of years. RABV infection leads to neurological symptoms and death; however, its pathogenesis in the brain is unclear, which complicates patient care. Given that no treatment exists for symptomatic cases, there is an urgent need for effective antiviral drugs. In this study, we aimed to investigate the pathogenic mechanism of RABV in the brain and screen for potential anti-RABV drugs. Protein samples were extracted from the brains of RABV-positive and RABV-negative dogs, and proteomic and phosphoproteomic analyses were conducted. The results showed that the synaptic vesicle cycle is critical to RABV pathogenesis. The kinases involved in the phosphorylation of proteins in the synaptic vesicle cycle were identified and examined as potential drug targets. Casein kinase 2 and protein kinase C were found to be key kinases for RABV replication, and five inhibitors of these enzymes were tested for their anti-RABV properties. Pretreating cells with the kinase inhibitor sunitinib significantly reduced the viral yield after RABV infection. Our findings suggest that RABV interferes with synaptic communication, which leads to rabies, and that inhibiting a vital kinase can reduce viral production. Hence, our findings have implications for the development of rabies treatment regimes.

## Introduction

Rabies is one of the deadliest diseases that humankind has ever experienced. Humans have been infected with the causative rabies virus (RABV) since ancient times, and rabies still causes approximately 60,000 deaths every year worldwide [1]. Children who live in countries where dog rabies is endemic are at the highest risk of RABV infection, and cases mostly occur in poor countries [2]. RABV is typically transmitted to humans from rabid animals via a bite. RABV virions present in the animal’s saliva enter the body via the bite wound and proceed to infect nearby myocytes before entering neurons through the neuromuscular junction. Virions then travel in a retrograde manner to the central nervous system and, finally, to the brain, the organ most affected by RABV infection. RABV infects different parts of the brain and causes inflammation and cerebral damage. It leads to two main clinical manifestations, which are categorized as encephalitic and paralytic rabies [3,4]. Patients with encephalitic rabies may experience fluctuating consciousness, hydrophobia, respiratory spasms, and seizures. Patients with paralytic rabies may experience flaccid limb weakness, paralysis, and urinary incontinence. The average incubation period is one to two months, and death is almost certain in patients who show signs of the disease [3].

When an RABV virion enters a neuron, it releases its genome of approximately 12,000 kilobases of RNA, and the genome is translated into five proteins (nucleoprotein, phosphoprotein, matrix protein, glycoprotein, and viral RNA polymerase). The viral proteins then perform their functions and create new virions [5]. Viral antigens tend to localize around the neuronal nucleus and along dendrites. Interestingly, RABV has been found to induce apoptosis in immune cells rather than neurons, and it has been hypothesized that this helps protect virions and their host cells from lysis [6].

The effects that RABV infection has on neurons at the molecular level are still unclear; however, there is a growing amount of research being conducted in this area. Proteins involved in several key cellular processes (e.g., apoptosis, immunoregulation, and metabolism) have been found to be differentially expressed in patients infected with RABV [7]. Similar findings were reported in a study conducted with brains from dogs infected with RABV; many proteins were found to be dysregulated, particularly proteins associated with calcium signaling and transport pathways [8]. In fact, altered intracellular signaling is one of the most commonly reported effects of RABV infection on neurons. In a study of mice infected with a pathogenic strain of RABV (challenge virus standard-11 [CVS-11]), a transcriptomic analysis indicated that many genes that encode proteins involved in signaling pathways, including the cyclic guanosine monophosphate–protein kinase G (cGMP-PKG) and mitogen-activated protein kinase (MAPK) signaling pathways, were affected by RABV infection [9]. In addition, intracerebral RABV infection in mice was shown to affect many signaling pathways in microglial cells, including the tumor necrosis factor (TNF), retinoic acid-inducible gene I (RIG-I), nucleotide-binding oligomerization domain (NOD), nuclear factor kappa-light-chain-enhancer of activated B cells (NF-κB), MAPK, and Janus kinase/signal transducers and activators of transcription (JAK-STAT) signaling pathways, and contribute to disease pathogenesis [10]. Notably, the phosphorylation of proteins involved in the MAPK and NF-κB signaling pathways, which play key roles in host responses to viral infections, was found to be elevated in the brains of naturally infected humans and dogs [11]. It has been suggested that increased protein phosphorylation is a marker of the activation of pathways that contribute to countering RABV infection, and this reflects the importance of protein modification in rabies progression [11].

Phosphorylation is one of the most important and common post-translational modifications of proteins. It often triggers intracellular communication and signaling cascades [12]. However, the impacts of phosphorylation, and the roles it plays, during RABV infection in the brain have not been fully elucidated. While studies have been conducted on RABV phosphoprotein and the phosphorylation of host proteins during infection with other viruses, only one study has focused on the phosphorylation of host proteins during RABV infection [11]. Such studies have highlighted that protein phosphorylation is a potential target for antiviral treatments. For instance, Lim et al. [13] conducted a phosphoproteomic analysis of hepatitis B virus-infected liver cell lines and found that the levels of 2,677 phosphoproteins were altered as a result of infection, as well as immune response, cell cycle process, and RNA processing pathways. Interestingly, pharmacological inhibition of a group of enzymes that catalyze phosphorylation (kinases) reduced the number of virus-infected cells [13]. In addition, Bouhaddou et al. performed global phosphoprotein profiling to investigate the impact of severe acute respiratory syndrome coronavirus 2 (SARS‑CoV‑2) infection and found that it activated many kinases, which led to cell cycle arrest, and that inhibiting the kinases significantly reduced the viral titer. Hence, kinase inhibitors have potential as antiviral agents [14].

Kinase inhibitors that are used to treat cancer have recently been found to be effective against many viral infections [15,16]. Repurposing drugs approved for treating other diseases as antiviral agents has several advantages. Costs can be reduced, and the development process can be accelerated. Moreover, targeting host machinery that is essential for viral replication may limit the risk of the development of drug resistance, which is a major challenge in the development of antiviral agents [17,18].

Therefore, in this study, we aimed to identify 1) the molecular impact that RABV has on the brain and 2) anti-RABV agents. To achieve this, we collected brain samples from dogs naturally infected with RABV and performed proteomic, phosphoproteomic, and bioinformatic analyses. We determined that the synaptic vesicle cycle is a key pathogenesis pathway of RABV, selected kinases involved in this cycle, and assessed the efficacy of kinase inhibitors as anti-RABV agents *in vitro.* Our findings suggest that kinase inhibitors have potential as therapeutic agents for the treatment of rabies, which is currently incurable. By developing rabies treatment protocols, it may be possible to reduce or even eliminate rabies-related mortality.

## Materials and methods

### Collection of brain samples from dogs with and without RABV infection

Brain samples were collected from dog carcasses sent to the Queen Saovabha Memorial Institute, Thai Red Cross Society, for the diagnosis of RABV infection. RABV infection was confirmed using an immunofluorescence assay (IFA). Cerebrum samples from three RABV-positive and three RABV-negative dogs were collected and stored at −80°C until further analysis.

### Protein extraction and phosphoprotein enrichment

Approximately 1 g of each brain sample was mixed with RIPA lysis buffer and homogenized using a Dounce homogenizer. The lysate was sonicated, and the debris was removed by centrifugation at 13,200 rpm for 20 min at 4°C. The protein concentration was measured using the Bradford assay.

The proteins in the RABV-positive and RABV-negative brain samples were subjected to phosphoprotein enrichment using the TALON PMAC Magnetic Phospho Enrichment Kit (Takara, Shiga, Japan) according to the manufacturer’s protocol. In brief, 500 µg of protein was added to metal ion-coated magnetic beads and incubated for 90 min in a rotary shaker at 4°C. The unbound proteins in the supernatant were discarded, and the beads were thoroughly washed. The phosphoproteins were eluted using 75 µL of the elution buffer. Phosphoprotein enrichment was confirmed using sodium dodecyl sulfate-polyacrylamide gel electrophoresis (SDS-PAGE) and Pro-Q diamond staining (Thermo Fisher Scientific, MA, USA).

### Protein separation and in-gel tryptic digestion

For the proteomic analysis, 100 µg of protein was subjected to 12% SDS-PAGE, and the separated proteins were stained with Coomassie Blue G. Protein bands were excised from the gel and subjected to in-gel digestion. For the phosphoproteomic analysis, 20 µL of the eluted sample was subjected to separation, and the separated phosphoproteins were stained with silver. Similarly, protein bands were excised and subjected to in-gel digestion.

The Coomassie dye was removed from the gel pieces by incubation with 25 mM ammonium bicarbonate in 50% acetonitrile. The silver was removed by incubation with 30 mM potassium ferricyanide and 156 mM sodium thiosulfate at a 1:1 ratio. Proteins were reduced with 4 mM dithiothreitol and alkylated with 250 mM iodoacetamide. Gel pieces were dehydrated with 100% acetonitrile, and then 10 ng trypsin was applied overnight to break the proteins into peptides. The peptides were extracted by adding 200 µL of acetonitrile. The eluted peptides were transferred to a new tube, lyophilized, and stored at −80°C.

### Mass spectrometry

Peptides were dissolved in 0.1% formic acid and then analyzed using an UltiMate 3000 nano-liquid chromatography system (Thermo Fisher Scientific) coupled to a micrOTOF-Q electrospray ionization quadrupole time-of-flight mass spectrometer (Bruker Daltonics, MA, USA). Mobile phase A consisted of 2% acetonitrile and 0.1% formic acid in water, and 0.1% formic acid in acetonitrile was used as mobile phase B. The flow rate was 300 nL/min, and the elution time was 60 min. MS data were acquired using Hystar software (Bruker Daltonics), and the mass ranges were *m/z* 400–3,000 and 50–1,500.

### Data and bioinformatics analyses

Protein annotation was performed using the Mascot Daemon software program (Matrix Science, London, UK). In brief, the data from all the gel pieces were merged into a single mascot generic file (.mgf) and then compared with the dog (*Canis lupus*) taxonomy. Only one missed cleavage was allowed, and no fixed modification was selected. Several variable modifications were selected, including methionine oxidation, cysteine carbamidomethylation, serine phosphorylation, tyrosine phosphorylation, and threonine phosphorylation. Peptide tolerance was assigned a value of 200 ppm, and the tandem MS tolerance was assigned a value of 0.6 Da. Proteins that were present in at least two of three replicates and showed at least 2-fold differential expression or phosphorylation were selected for further analysis.

A gene ontology analysis was performed using the PANTHER classification system (https://www.pantherdb.org/) on January 20, 2023 [19]. The list of differentially expressed and phosphorylated proteins was added to the software, and the organism *Canis lupus familiaris* was selected. Data were analyzed against the categories of molecular function, biological process, and cellular components.

A KEGG pathway analysis was performed using STRING (https://string-db.org/) on January 17, 2023 [20,21]. The list of differentially expressed and phosphorylated proteins was added to the software, and the organism *Canis lupus familiaris* was selected. The full STRING network type was selected with medium confidence (0.400). No “maximum number of interactions to show” value was chosen for both the first and second shells.

### Identification of phosphorylation sites and kinases

The kinase identification was performed using NetPhos – 3.1 (https://services.healthtech.dtu.dk/services/NetPhos-3.1/) on January 20, 2023 [22]. The sequences of SYT1 (P21579), MUNC18 (P61764), and RABV phosphoprotein (P22363) were retrieved from the UniProt database [23]. The phosphorylation sites and corresponding kinases were identified for all three phosphorylated amino acids. Only phosphorylation sites with scores > 0.5 were considered positive.

### Cell and virus cultures

The RABV CVS-11 strain was provided by the Department of Medical Services, Ministry of Public Health, Thailand. The virus was cultured in mouse neuroblastoma (Neuro-2a) cells. The cells were cultured in Eagle’s Minimum Essential Medium supplemented with 10% fetal bovine serum and penicillin–streptomycin. A viral titration was performed using an IFA, and the titer was calculated using Reed and Muench’s formula [24].

### Cell viability assay

The kinase inhibitors sunitinib malate, silmitasertib, chelerythrine chloride (Sigma-Aldrich, MO, USA), rottlerin, and DMAT (MedChemExpress, NJ, USA) were purchased from companies and dissolved in dimethyl sulfoxide (DMSO). They were diluted to the desired concentrations (10 µM, 7.5 µM, 5 µM, 2.5 µM, 1 µM, 100 nM, and 10 nM) with cell culture medium. The medium containing the inhibitors was added to Neuro-2a cells, and the cells were monitored for morphological changes at 24, 48, and 72 h. At 72 h post-treatment, cell viability was assessed using PrestoBlue^TM^ (Invitrogen, MA, USA). The reagent was added to the medium, and the cells were incubated for 1 h. The absorbance was measured at 570 nm.

### Detection of protein phosphorylation by western blot

The effects that each kinase inhibitor had on protein phosphorylation were evaluated via western blot analysis. Neuro-2a cells were cultured and treated with 7.5 µM sunitinib malate, 7.5 µM silmitasertib, 7.5 µM chelerythrine chloride, 7.5 µM rottlerin, or 10 µM DMAT for 0, 16, 24, 48, and 72 h. Proteins were extracted, the protein concentration was determined, and 30 µg of protein was subjected to western blot analysis. Anti-phosphoserine antibody (Sigma-Aldrich) was used, and the chemiluminescence substrate (Thermo Fisher Scientific) was used to detect the immunoreactive bands.

### *In vitro* inhibition of RABV replication

To test the effect of the kinase inhibitors on RABV replication, Neuro-2a cells were cultured and treated with 7.5 µM sunitinib malate, 7.5 µM silmitasertib, 7.5 µM chelerythrine chloride, 7.5 µM rottlerin, or 10 µM DMAT for 48 h, and DMSO was used as the vehicle control. Cells were then exposed to RABV at 0.01 MOI for 90 min before the free virions were washed away. Supernatant samples were collected at 24, 48, and 72 h post-infection. The virus present was titrated using an IFA, and the levels were compared across the test and control samples. The focus-forming dose 50 (FFD_50_)/mL value from each sample was standardized with the value at the time of infection, and then the percentage of inhibition was calculated by comparing the test and control values.

### Statistical analyses

All statistical analyses were performed using the RStudio package. The Shapiro–Wilk test was used to assess normality. The t-test was applied to normally distributed data, while the Mann–Whitney test was applied to non-normally distributed data. When *p* was < 0.05, the result was deemed significant.

## Results

### Protein and phosphoprotein profiles of brain samples from RABV-infected dogs

**Fig 1:**
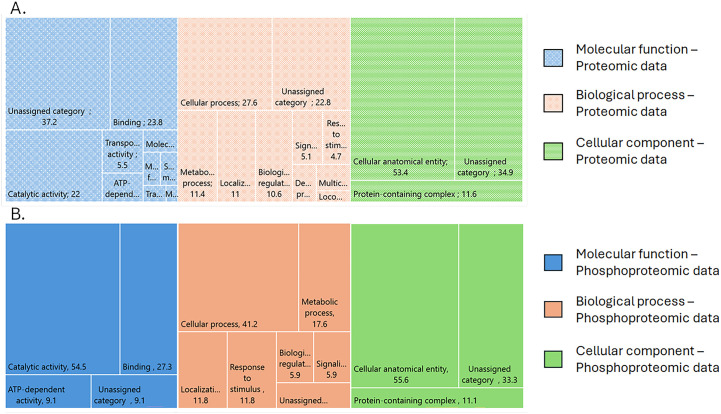
Gene ontology analysis results for the proteomic and phosphoproteomic data. A. Results obtained using the proteomic data. B. Results obtained using the phosphoproteomic data.

Among the 5,933 proteins identified in the brain samples from RABV-positive and RABV-negative dogs, 255 were differentially expressed: 150 proteins were upregulated and 105 proteins were downregulated (Tables 1 and S1). Our gene ontology analysis indicated that among the differentially expressed proteins with molecular functions, 37.2%, 23.8%, and 22% were involved in unassigned activities, binding, and catalytic activity, respectively. For those involved in biological processes, the top three types of processes were cellular (27.6%), unassigned (22.8%), and metabolic (11.4%) processes. The proteins that were classified as cellular components fell into three categories: cellular anatomical entity (53.4%), unassigned entity (34.9%), and protein-containing complex (11.6%) (Fig 1A, S2 Table).

**Table 1:** Top 10 proteins differentially expressed in brain samples from dogs infected with rabies virus (vs. brain samples from dogs without rabies virus).

No.	Accession No.	Protein Name	MW	pI	Protein Score	Sequence Coverage	Average Fold-change
**Proteins with increased expression**							
1	F1PNP2_CANLF	Neurofilament heavy	125371	8.22	885	21.5	12.50
2	A0A5F4CM08_CANLF	Neurofilament light polypeptide	58869	4.83	1284	43.7	8.41
3	A0A5F4CY33_CANLF	Ubiquitin-activating enzyme E1	118775	5.8	475	24.5	6.78
4	A0A5F4C4V0_CANLF	Voltage-dependent anion-selective channel protein 1	24402	6.74	182	30.3	6.43
5	A0A5F4BVG8_CANLF	Alpha-1,4 glucan phosphorylase	95795	7.62	167	10.9	6.00
6	A0A5F4CY61_CANLF	2-phospho-D-glycerate hydrolyase	13561	9.61	124	29.3	5.84
7	E2RRM6_CANLF	Heat shock protein family A (Hsp70) member 4 like	107866	5.64	321	15	4.67
8	A0A5F4C6X4_CANLF	Dihydrolipoyl dehydrogenase	53282	7.21	147	14.6	4.50
9	A0A5F4C5N2_CANLF	Peroxiredoxin 6	25252	6.21	259	38.7	4.05
10	E2RQ14_CANLF	Annexin	47784	5.47	840	46	3.94
**Proteins with decreased expression**							
1	J9P8P9_CANLF	Calcium-transporting ATPase	133114	5.57	366	14.7	−4.33
2	A0A5F4CSY2_CANLF	Apolipoprotein E	36582	5.41	461	34.2	−4.18
3	A0A5F4DB38_CANLF	Peptidase S1 domain-containing protein	35574	5.87	418	41.6	−4.16
4	J9P7B8_CANLF	Synaptobrevin-2	12641	7.85	174	35.3	−3.89
5	K2C1_CANLF	Keratin, type II cytoskeletal 1	63751	7.66	203	17.4	−3.73
6	J9P540_CANLF	Glyceraldehyde-3-phosphate dehydrogenase	36141	7.16	308	35	−3.60
7	E2RPM2_CANLF	Synaptosomal-associated protein	23300	4.66	253	48.1	−3.55
8	E2RJQ8_CANLF	V-type proton ATPase subunit	40375	4.85	190	13.1	−3.38
9	F1PYU9_CANLF	Keratin, type I cytoskeletal 10	57650	5.09	264	13.4	−3.06
10	J9NTB4_CANLF	Uncharacterized protein	29385	8.89	140	12.5	−3.03

Likewise, the phosphoproteomic analysis results revealed that there were 41 differentially phosphorylated proteins. Phosphorylation was increased in 32 proteins and decreased in seven proteins (Tables 2 and S3). The gene ontology analysis of the differentially phosphorylated proteins showed that the proteins with molecular functions were mainly involved in catalytic activity (54.5%) and binding (27.3%). For the proteins involved in biological processes, cellular processes (41.2%) and metabolic processes (17.6%) were the top categories. For the proteins that were classified as cellular components, the phosphoproteomic findings were similar to the proteomic findings, with the proteins mainly classified as cellular anatomical entities and unassigned entities (Fig 1B, S2 Table).

**Table 2:** Top 10 proteins differentially phosphorylated in brain samples from dogs infected with rabies virus (vs. brain samples from dogs without rabies virus).

No.	Accession No.	Protein Name	MW	pI	Protein Score	Sequence Coverage	Average Fold-change
**Proteins with increased phosphorylation**							
1	KCRB_CANLF	Creatine phosphokinase M-type	42674	5.47	59	2.9	5.53
2	XP_005619707.1	Heat shock cognate 71 kDa protein	70854	5.37	93	4.5	5.00
3	XP_849125.1	Dihydropyrimidinase-related protein 2 isoform X1	73545	5.98	99	8	4.52
4	XP_022262693.1	Peroxiredoxin-2 isoform X1	26016	5.69	72	20.1	2.08
5	RCN39677.1	Hypothetical protein ANCCAN_14376	70387	5.36	57	4.5	2.00
6	XP_038474787.1	Syntaxin-binding protein 1 isoform X1	68692	6.32	121	8.5	2.00
7	ANW72321.1	Mitochondrial ATP synthase subunit alpha subunit	59691	9.22	156	11.2	- *
8	ANW72322.1	Mitochondrial ATP synthase subunit beta subunit	56250	5.21	111	15.6	- *
9	AQX24407.1	Molecular chaperone DnaK	68202	4.85	94	9.2	- *
10	AUZ82886.1	Immunoglobulin heavy chain variable region, partial	13625	4.96	75	32.3	- *
**Proteins with decreased phosphorylation**							
1	AAA30879.1	Beta-spectrin, partial	81069	5.47	143	4.8	−3.25
2	XP_005626165.1	Spectrin beta chain, non-erythrocytic 1 isoform X2	272632	5.44	541	5.4	−2.25
3	BAR79439.1	Annexin A5	35921	4.99	107	8.1	−2.00
4	AUZ82936.1	Immunoglobulin heavy chain variable region, partial	13643	4.98	86	36.8	0.00
5	CAD10571.1	Unnamed protein product	51926	8.52	258	12.7	0.00
6	NP_001332949.1	Keratin, type II cytoskeletal 2 oral	66237	8.78	122	16.5	0.00
7	RCN29942.1	Hypothetical protein ANCCAN_24286, partial	103913	5.81	48	5.2	0.00

* Protein was only detected in brain samples from dogs infected with rabies virus.

### The synaptic vesicle cycle was implicated in RABV pathogenesis

Next, we used the proteomic data to identify molecular pathways potentially associated with the pathogenesis of RABV infection. A protein interaction network analysis performed using STRING revealed that the protein–protein interaction enrichment *p-*value was < 1.0e^-16^, which indicated a biological connection between the differentially expressed proteins. In addition, 30 pathways were identified from our proteomic data in a Kyoto Encyclopedia of Genes and Genomes (KEGG) pathway enrichment analysis (S4 Table).

**Fig 2:**
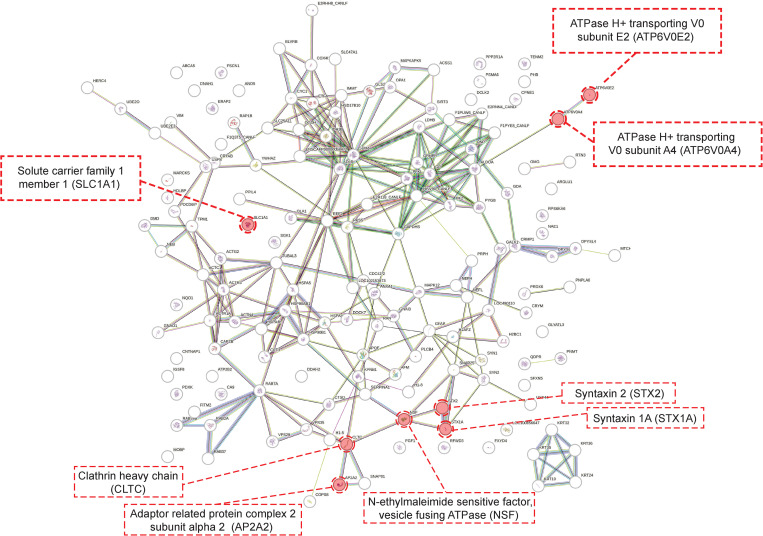
The analysis of protein-protein interaction network from proteomic results. The results of the STRING analysis performed with the differentially expressed proteins. The red circles indicate proteins involved in the synaptic vesicle cycle.

**Fig 3:**
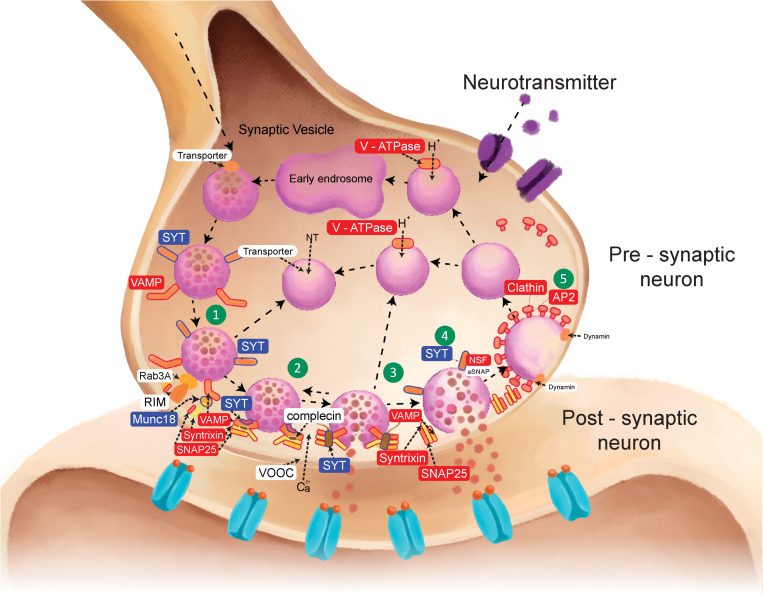
The differentially expressed and phosphorylated proteins associated with the synaptic vesicle cycle. The synaptic vesicle cycle, adapted from the Kyoto Encyclopedia of Genes and Genomes database (pathway entry: cfa04721), and its five steps: docking (1), priming (2), fusion (3), full fusion (4), and endocytosis (5). The red rectangles indicate differentially expressed proteins, and the blue rectangles indicate differentially phosphorylated proteins.

Interestingly, the synaptic vesicle cycle was enriched, with a strength of 1.2 and a false discovery rate of 7.27e^-06^. It has been shown that synaptic transportation is the major mode of transmission used by RABV within the nervous system, and altered expression of genes and proteins involved in the synaptic vesicle cycle has been reported. In the STRING analysis, eight nodes were associated with the synaptic vesicle cycle (Fig 2), and the KEGG analysis showed that 12 of the differentially expressed proteins and two of the differentially phosphorylated proteins were associated with the synaptic vesicle cycle (Fig 3, Table 3). Most of the identified proteins are involved in the release of neurotransmitters into the synaptic cleft, which is a vital process for RABV transmission. Changes in the expression and phosphorylation of proteins involved in this process may affect the ability of RABV virions to infect neurons and significantly influence the pathogenesis of rabies. Therefore, we explored the possibility of identifying anti-RABV drugs based on their effects on the phosphorylation of proteins involved in the synaptic vesicle cycle.

**Table 3:** Differentially expressed and phosphorylated proteins involved in the synaptic vesicle cycle.

No.	KEGG ID	KEGG Name	Accession No.	Protein Name	Protein Feature Change	Level
1	K06027	*N*-ethylmaleimide sensitive factor, vesicle-fusing ATPase (NSF)	E2RFV4_CANLF	Vesicle-fusing ATPase	Expression	2.58
2	K04646	Clathrin heavy chain (CLTC)	A0A5F4C5Z1_CANLF	Clathrin heavy chain	Expression	2.58
3			A0A5F4D6L0_CANLF	Clathrin heavy chain	Expression	2.48
4	K11824	Adaptor related protein complex 2 subunit alpha 1 (AP2A1)	F1PE67_CANLF	AP-2 complex subunit alpha	Expression	2.08
5	K08486	Syntaxin 2 (STX2)	F1PR71_CANLF	Syntaxin 2	Expression	- *
6			A0A5F4BY58_CANLF	Syntaxin 2	Expression	0
7	K02146	ATPase H^+^ transporting V0 subunit d1 (ATP6V0D1)	A0A5F4BNZ8_CANLF	V-type proton ATPase subunit a	Expression	0
8			F1PVS8_CANLF	V-type proton ATPase subunit a	Expression	−2.70
9	K04560	Syntaxin 1A (STX1A)	F1PVP1_CANLF	Syntaxin 1A	Expression	−2.22
10	K18211	Synaptosome associated protein 25 (SNAP25)	E2R907_CANLF	Synaptosomal-associated protein	Expression	−2.63
11			E2RPM2_CANLF	Synaptosomal-associated protein	Expression	−3.57
12	K13504	Vesicle associated membrane protein 2 (VAMP2)	J9P7B8_CANLF	Synaptobrevin-2	Expression	−3.84
13	K15292	Syntaxin-binding protein 1 (MUNC18)	XP_038474787.1	Syntaxin-binding protein 1 isoform X1	Phosphorylation	2
14	K15290	Synaptotagmin 1 (SYT1)	XP_038305255.1	Synaptotagmin-1 isoform X1	Phosphorylation	- *

* Protein was only detected in brain samples from dogs infected with rabies virus.

### Casein kinase 2 and protein kinase C were identified as potential targets of anti-RBV treatments

**Fig 4:**
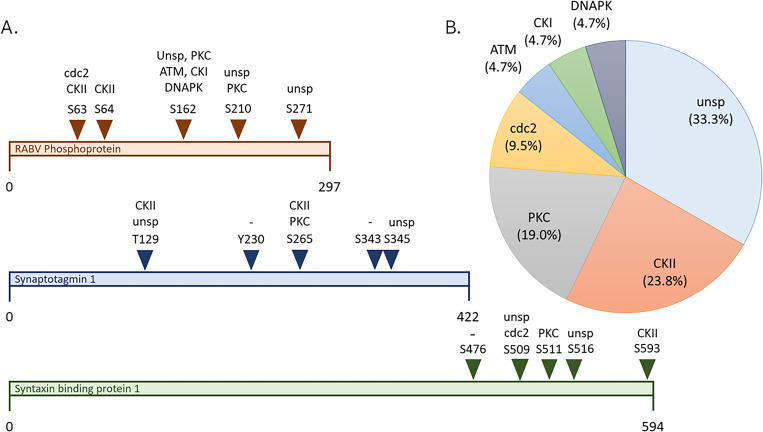
Identification of kinases involved in the phosphorylation of RABV phosphoprotein, synaptotagmin-1, and syntaxin-binding protein 1. A. Schematic diagram showing the phosphorylation sites and their corresponding kinases. B. Pie chart showing the percentage of phosphorylation sites on the target proteins associated with each kinase.

In this study, we worked to determine the phosphorylation sites and corresponding kinases of three proteins: RABV phosphoprotein, syntaxin-binding protein 1 (MUNC18), and synaptotagmin-1 (SYT1). RABV phosphoprotein is vital for viral replication, and inhibition of its phosphorylation significantly affects its function [25,26]. Likewise, MUNC18 and SYT1 are involved in the synaptic vesicle cycle and were found to be differentially phosphorylated. We hypothesized that phosphorylation of MUNC18 and SYT1 is essential for RABV replication and that altered phosphorylation of these proteins reduces viral replication. We determined that there are five phosphorylation sites on each of RABV phosphoprotein, MUNC18, and SYT1 (Fig 4A). Regarding the kinases involved in the phosphorylation of the three proteins, in most cases, the kinase was not specified; however, casein kinase 2 (CKII) and protein kinase C (PKC) were identified as the second- and third-most commonly acting kinases (Fig 4B). Thus, we next searched for CKII and PKC inhibitors and tested a selection for their anti-RABV potential in an *in vitro* assay. Kinases have previously been proposed as targets of antiviral drugs.

### Kinase inhibitor selection and anti-RABV activity in vitro

The following criteria were used to select the kinase inhibitors to be assessed for anti-RABV activity: 1) targets CKII or PKC and/or 2) shows activity against RABV or other viruses. The drugs selected for testing were sunitinib, silmitasertib, chelerythrine, rottlerin, and 2-dimethylamino-4,5,6,7-tetrabromo-1H-benzimidazole (DMAT), and Neuro-2a cells were used in the *in vitro* experiments.

First, the concentrations of the drugs that resulted in ≤ 80% cell viability were determined, and these concentrations were not included in the study. Sunitinib, silmitasertib, and chelerythrine were cytotoxic at 7.5–10 µM. In contrast, rottlerin and DMAT did not affect cell viability at concentrations lower than 100 µM; hence, these drugs were tested at concentrations that were comparable to those of the other three drugs (S1 Fig). We then analyzed the effect that each kinase inhibitor had on protein phosphorylation. Protein phosphorylation was evaluated at five time points: before treatment and 16, 24, 48, and 72 h after treatment. All the kinase inhibitors effectively reduced the level of protein phosphorylation, and the largest declines (compared to before treatment) were observed at 48 h post-treatment (S2 Fig).

**Fig 5:**
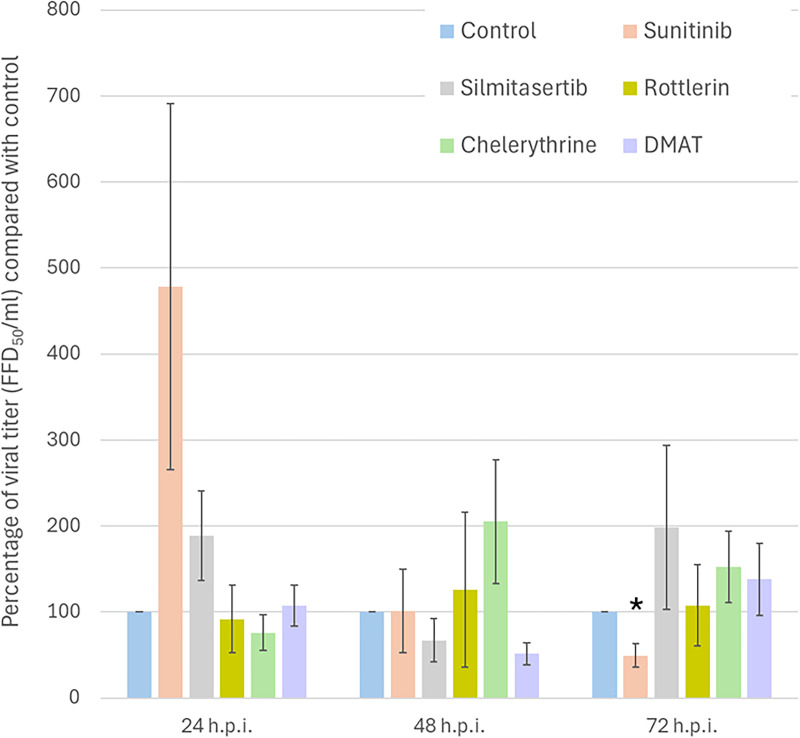
Rabies virus production in kinase inhibitor-treated Neuro-2a cells at three time points. Data are shown as mean ± standard error. * *p* < 0.05. hpi: hours post-infection.

To observe the impact that the drug-induced reduction in phosphorylation had on viral replication, Neuro-2a cells were pretreated with each drug for 48 h and then infected with RABV. The number of new virions was measured 24, 48, and 72 h after infection. Pretreating the cells with the kinase inhibitors mostly resulted in non-significant fluctuations in the virus level. However, pretreatment with sunitinib for 48 h significantly decreased the level of RABV at 72 h post-infection to effectively half (0.49-fold) that of the control, indicating that this drug had a profound effect on virus replication (Fig 5). Hence, sunitinib has potential as an anti-RABV agent.

## Discussion

RABV has posed a threat to human health for thousands of years, and even though a vaccine has been developed, there is no treatment available for symptomatic RABV infection, and patients continue to die from rabies. In this study, we performed proteomic and phosphoproteomic analyses of brain samples from dogs naturally infected with RABV to identify the pathogenic pathway of RABV. The synaptic vesicle cycle was found to be a key factor in RABV infection. We used bioinformatic tools to identify potential druggable targets in this cellular process, and our results showed that CKII and PKC play vital roles in the phosphorylation of proteins involved in the synaptic vesicle cycle. Finally, we performed a series of *in vitro* experiments with five kinase inhibitors and found that sunitinib could effectively reduce RABV replication.

The use of proteomic and phosphoproteomic methods to elucidate the pathogenesis of viral diseases is well established. For example, Giansanti et al. [27] used proteomic and phosphoproteomic methods to examine the alterations caused by coxsackievirus in an *in vitro* human cell model. They found changes in 798 proteins and detected 16,944 phosphorylation sites in > 3,500 proteins. The mammalian Target of Rapamycin Complex 1 (mTORC1) signaling pathway was identified as the key regulator of viral infection; inhibiting this pathway activated transcription factor EB, which promoted the release of coxsackievirus virions through extracellular vesicles. Their findings demonstrated that proteomics and phosphoproteomics could be used to enhance our understanding of viral replication. Proteomic and phosphoproteomic investigation has been performed in a number of viral diseases, for instance, SARS-CoV-2 [28], influenza A virus [29], hepatitis B virus [30]. However, no studies have yet focused on RABV infection. Our proteomic and phosphoproteomic results provide new insights into the mechanisms used by RABV to infect neurons and replicate. By identifying proteins that are differentially expressed and phosphorylated in RABV-infected cells, we revealed many pathways potentially involved in the pathogenesis of RABV. The top three pathways, with the highest strength values, are involved in cellular metabolism (S4 Table). The identified cellular metabolism pathways are essential for cell survival; however, they lack specificity to RABV infection. Hence, we focused on the synaptic vesicle cycle because RABV virions are known to spread between neurons via synaptic transportation [31].

The synaptic vesicle cycle regulates the release and recycling of vesicles used in neurotransmitter communication between neurons. Vesicles containing neurotransmitters are docked in the active zone, then MUNC18 in this area binds to SYT1 on the surface of the synaptic vesicles (Fig 3, number 1). During this step, several proteins, including VAMP2, STX1, and SNAP25, assemble to form the *N*-ethylmaleimide sensitive factor attachment protein receptor (SNARE) complex (Fig 3, number 2). When the nerve impulse arrives, Ca^2+^ influx occurs and triggers the fusion of the vesicles to the plasma membrane through a conformational change in the SNARE complex (Fig 3, number 3). After the neurotransmitters are released, *N*-ethylmaleimide-sensitive factor (NSF) and α-SNAP proteins disassemble the complex (Fig 3, numbers 4–5). The portion of each vesicle that fuses with the plasma membrane is recycled by binding to CLTR and AP2 (Fig 3, number 5) and undergo several processes for preparation of the new cycles [32,33]. The perturbation of the synaptic vesicle cycle and its protein machinery have been linked to neurological diseases, including Alzheimer’s disease and Parkinson’s disease [34,35]. Interestingly, changes to proteins involved in this cycle (e.g., VAMP2, synapsin 2, and transmembrane protein TMEM230) have been associated with seizures, one of the main clinical outcomes of RABV infection, in both animal and human subjects [36–38]. Regarding RABV infection of neurons, there is considerable evidence that both mRNAs and proteins that play crucial roles in the synaptic vesicle cycle are dysregulated by the virus [9,39–42]. Notably, our mass spectrometry (MS) analysis showed that there were changes in the expression and phosphorylation of proteins involved in this process, for example, MUNC18, SYT1, VAMP2, STX1, SNAP25, CLTC, and AP2A1 (Table 3). Based on the above findings, we hypothesized that RABV interferes with synaptic transportation in the brain by altering protein expression and phosphorylation, which impairs neuronal signaling and causes the clinical symptoms of RABV infection observed in animals and humans.

MUNC18 and SYT1 were selected for further analysis because they were differentially phosphorylated after RABV infection, which indicates that they may play a role in viral replication. Likewise, RABV phosphoprotein was selected for further analysis because it is consistently phosphorylated and its phosphorylation is essential for viral replication [25,26]. We used information about the phosphorylation sites on MUNC18, SYT1, and RABV phosphoprotein to identify the corresponding kinases. In most cases, the kinases involved in the phosphorylation of these proteins could not be specified; however, CKII and PKC were identified as kinases involved in the phosphorylation of MUNC18, SYT1, and RABV phosphoprotein (Fig 4B). We then conducted a literature search to identify CKII, PKC, and RABV inhibitors. CKII is a serine/threonine kinase involved in the phosphorylation of multiple target proteins. CKII has been proposed as a drug target for the treatment of many human diseases, including viral infections [43]. For example, CKII was found to phosphorylate the nonstructural 5A protein of the hepatitis C virus, a key protein for viral RNA replication [44]. Moreover, nonstructural protein 1 of rotavirus is reportedly phosphorylated by CKII, leading to host immune evasion [45]. PKC is also a serine/threonine kinase. This lipid-dependent enzyme triggers signal transduction in many processes, including apoptosis, cell differentiation, and angiogenesis [46]. PKC activity has also been associated with many viral diseases. For example, it has been shown that inhibiting PKC significantly reduces SARS-CoV-2 replication in the early stages of infection [47]. Intriguingly, PKC was found to be involved in the phosphorylation of RABV phosphoprotein, facilitating its nuclear localization [25]. These pieces of evidence suggest that CKII and PKC are essential for many viruses to maintain their normal functions and that drugs designed to inhibit CKII and PKC may have antiviral activity. Therefore, we searched for kinase inhibitors to test for anti-RABV activity. Based on the search results, we selected five drugs for *in vitro* anti-RABV activity testing: sunitinib [48], silmitasertib [49], chelerythrine [50], rottlerin [51], and DMAT [52].

All five drugs demonstrated the capacity to potently reduce overall phosphorylation (S2 Fig); however, only sunitinib showed anti-RABV activity (Fig 4). Sunitinib is a multikinase receptor tyrosine kinase reversible inhibitor. It inhibits phosphorylation by competitively binding to the ATP pocket of kinases. Evidence suggests that sunitinib is a broad-spectrum kinase inhibitor, and it has been shown to target CKII and PKC [53,54]. Sunitinib has shown its potential as an antiviral agent against many viruses. For example, Lin et al. [55] tested the antiviral efficacy and mechanisms of sunitinib in a series of experiments with the Zika virus. Their findings indicated that sunitinib could reduce the cytopathic effect, expression of viral proteins, and viral yield at relatively low concentrations. Moreover, they found that sunitinib exerted its activity in both the entry and post-entry phases of cellular infection. They proposed sunitinib as a potential anti-Zika drug. In another virus-based study, Tongmuang et al. [56] treated dengue virus-infected cells with sunitinib to investigate the impact of AP-2 phosphorylation on virus production. The phosphorylation of AP-2 is mediated by AP-2-associated protein kinase 1 (AAK1), and sunitinib is known to inhibit AAK1. They observed a consistent reduction in extracellular virions across all dengue virus serotypes after sunitinib treatment. Hence, their findings indicated that kinase inhibitors can have profound effects on virus production. In line with Tongmuang et al.’s [56] results, Luo et al. [57] found that RABV infection increased AAK1 expression at both the mRNA and protein levels. They also found that knocking down AAK1 significantly reduced RABV infection, while knocking out the AAK1 gene had a greater impact on the virus. Likewise, inhibiting AAK1 with sunitinib was shown to reduce RABV infection. Unfortunately, treating RABV-challenged mice with sunitinib did not improve the survival time. In contrast, Wang et al. [48] reported that treating RABV-infected mice with sunitinib prolonged their survival by six days. Their other findings align with those of Luo et al. [57]. They identified AAK1 as a crucial component for RABV entry into host cells through an RNA interference approach, and knocking down AAK1 limited the phosphorylation of AP-2 and decreased RABV infection *in vitro.* Moreover, sunitinib treatment reduced AP-2 phosphorylation and prevented RABV from entering the early endosomes. Despite conflicting findings about the effects of sunitinib in animal models, the phosphorylation of AP-2—which is mediated by AAK1—has been proven to be critical for RABV infection. AP-2 is a crucial component of the synaptic vesicle cycle and was found to be upregulated in our study (Table 3). Therefore, our findings suggest that the synaptic vesicle cycle is a key factor in RABV pathogenesis and that repurposing a kinase inhibitor that targets a component of the pathway is a potential strategy for treating RABV infection.

Although we successfully identified pathogenesis pathway of RABV infection in the brain and proposed a kinase inhibitor as the potential anti-RABV drug, there are some limitations remaining. Firstly, we assured that kinase inhibitor treatment led to reduction of overall protein phosphorylation, however, we did not confirm specifically to the phosphorylation level of the target phosphoproteins, MUNC18, SYT1, and RABV phosphoprotein. This limitation leaves a gap in understanding the relationship between phosphorylation levels of target proteins and RABV replication. Secondly, we predicted that CKII and PKC are the primary kinases involved in the phosphorylation of target proteins. We then treated the samples with kinase inhibitors to reduce their activities but did not measure the extent of activity reduction after treatment. In addition, kinase inhibitor drugs usually have broad range of targets. Hence, it raises the possibility that the reduction in RABV production may result from off-target effects. Further studies may focus on elucidating the impact of MUNC18 and SYT1 phosphorylation on RABV replication as well as confirmed the mechanisms of sunitinib as an anti-RABV agent.

## Conclusions

In conclusion, our findings indicate that the synaptic vesicle cycle is a vital part of the pathogenic pathway of RABV infection and that the kinase inhibitor sunitinib has potential as a repurposed antiviral agent due to its ability to significantly reduce RABV production *in vitro*. Hence, our findings provide valuable insight into how RABV causes disease in the brain and have implications for the development of an effective rabies treatment that could reduce or even eliminate deaths caused by RABV.

## Supporting information

S1 Fig Cell viability test of Neuro-2a cells after 72 hours of treatment with kinase inhibitor drugs. A. Sunitinib. B. Silmitasertib. C. Chelerythrine. D. Rottlerin. and E. DMAT. Data points represent mean ± S.D. * means p < 0.05, ** means p < 0.01.
(TIF)

S2 Fig Effect of kinase inhibitor drugs on overall protein phosphorylation. Neuro-2a cells were treated with non-toxic dose of each kinase inhibitor at 5 different time points. The overall phosphorylation level was investigated using western blot analysis with anti-phosphoserine antibody. A. Sunitinib. B. Silmitasertib. C. Chelerythrine. D. Rottlerin. and E. DMAT.
(TIF)

S1 Table Differentially expressed proteins from RABV-positive dog brains, in comparison with RABV-negative brains. (DOCX)

S2 Table Gene ontology analysis of proteomic and phosphoproteomic data. (DOCX)

S3 Table Differentially phosphorylated proteins from RABV-positive dog brains, in comparison with RABV-negative brains. (DOCX)

S4 Table Pathway analysis of differentially expressed proteins according to KEGG pathway. (DOCX)

S1 File S1_raw_images. (PDF)
