# Supplementary figures and images for: Proteomic and phosphoproteomic analysis of rabies pathogenesis in the clinical canine brain and identification of a kinase inhibitor as a potential repurposed antiviral agent
Source: PLoS One. 2025 Jun 27;20(6):e0323931. doi: 10.1371/journal.pone.0323931 (PMC12204518; doi:10.1371/journal.pone.0323931)

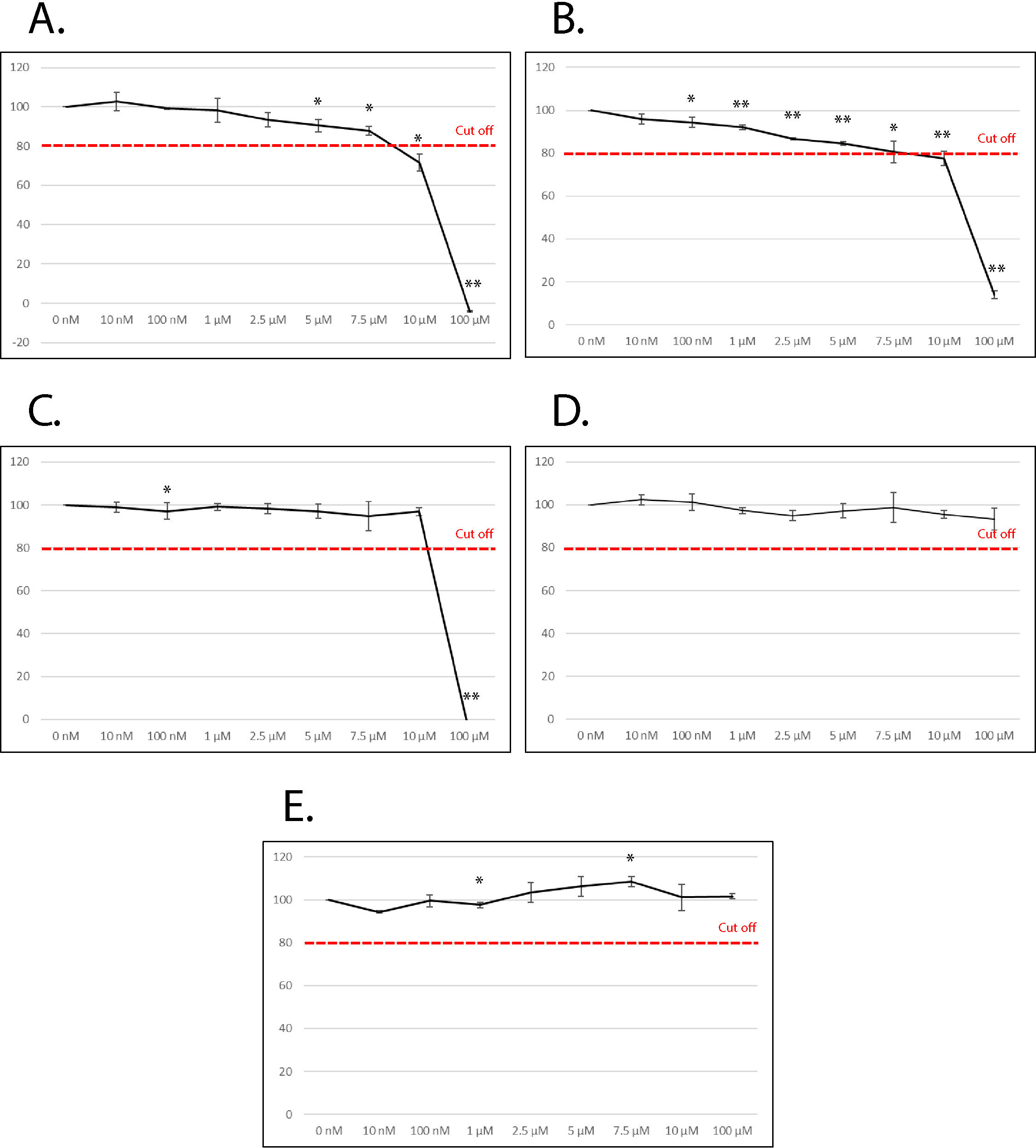

Supplement: S1 Fig — A. Sunitinib. B. Silmitasertib. C. Chelerythrine. D. Rottlerin. and E. DMAT. Data points represent mean ± S.D. * means p < 0.05, ** means p < 0.01. (TIF) [file pone.0323931.s001.tif]

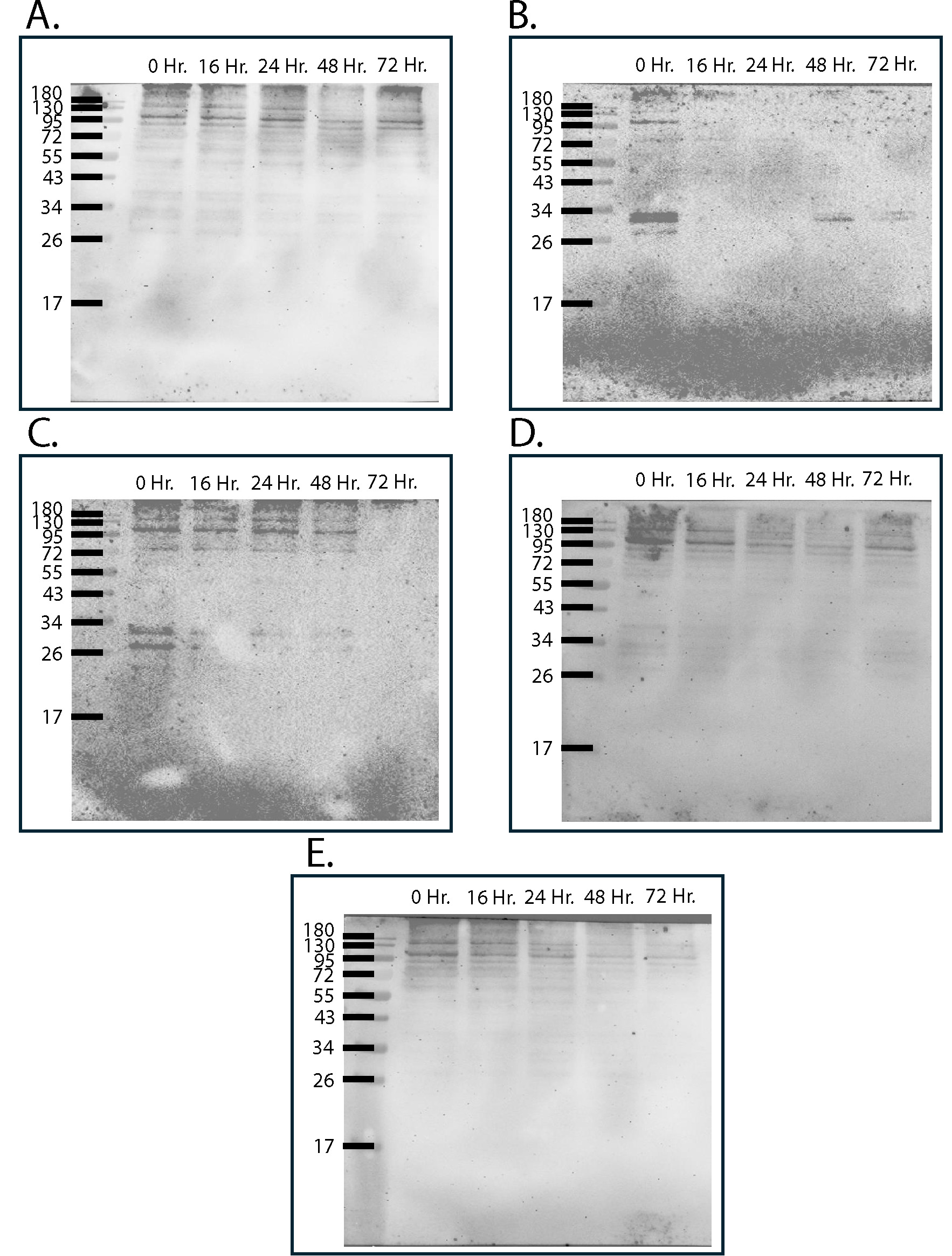

Supplement: S2 Fig — Neuro-2a cells were treated with non-toxic dose of each kinase inhibitor at 5 different time points. The overall phosphorylation level was investigated using western blot analysis with anti-phosphoserine antibody. A. Sunitinib. B. Silmitasertib. C. Chelerythrine. D. Rottlerin. and E. DMAT. (TIF) [file pone.0323931.s002.tif]
