# Supplementary material for: Proteomic and phosphoproteomic analysis of rabies pathogenesis in the clinical canine brain and identification of a kinase inhibitor as a potential repurposed antiviral agent
Source: PLoS One. 2025 Jun 27;20(6):e0323931. doi: 10.1371/journal.pone.0323931 (PMC12204518; doi:10.1371/journal.pone.0323931)
Supplement: S1 Table — (DOCX) [file pone.0323931.s003.docx]

**Table S1 Differentially expressed proteins from** **RABV-positive dog brains, in comparison with RABV-negative brains.**

| **No.** | **Accession No.** | **Protein Name** | **MW** | **pI** | **Protein Score** | **Sequence Coverage** | **Average Fold-change** |
| --- | --- | --- | --- | --- | --- | --- | --- |
| 1 | F1PNP2_CANLF | Neurofilament heavy | 125371 | 8.22 | 885 | 21.5 | 12.50 |
| 2 | A0A5F4CM08_CANLF | Neurofilament light polypeptide | 58869 | 4.83 | 1284 | 43.7 | 8.41 |
| 3 | A0A5F4CY33_CANLF | Ubiquitin-activating enzyme E1 | 118775 | 5.8 | 475 | 24.5 | 6.78 |
| 4 | A0A5F4C4V0_CANLF | Voltage-dependent anion-selective channel protein 1 | 24402 | 6.74 | 182 | 30.3 | 6.43 |
| 5 | A0A5F4BVG8_CANLF | Alpha-1,4 glucan phosphorylase | 95795 | 7.62 | 167 | 10.9 | 6.00 |
| 6 | A0A5F4CY61_CANLF | 2-phospho-D-glycerate hydrolyase | 13561 | 9.61 | 124 | 29.3 | 5.84 |
| 7 | E2RRM6_CANLF | Heat shock protein family A (Hsp70) member 4 like | 107866 | 5.64 | 321 | 15 | 4.67 |
| 8 | A0A5F4C6X4_CANLF | Dihydrolipoyl dehydrogenase | 53282 | 7.21 | 147 | 14.6 | 4.50 |
| 9 | A0A5F4C5N2_CANLF | Peroxiredoxin 6 | 25252 | 6.21 | 259 | 38.7 | 4.05 |
| 10 | E2RQ14_CANLF | Annexin | 47784 | 5.47 | 840 | 46 | 3.94 |
| 11 | J9NYL8_CANLF | GST class-pi | 23821 | 5.24 | 255 | 33.8 | 3.93 |
| 12 | A0A5F4D8M8_CANLF | MICOS complex subunit MIC60 | 75620 | 6.51 | 203 | 27 | 3.50 |
| 13 | F1P7M0_CANLF | Aminopeptidase | 96919 | 5.34 | 123 | 10.8 | 3.31 |
| 14 | J9NUZ0_CANLF | Dynamin GTPase | 95157 | 6.29 | 715 | 23.7 | 3.26 |
| 15 | CATD_CANLF | Cathepsin D | 44292 | 7.97 | 278 | 31.2 | 3.15 |
| 16 | F1PGY1_CANLF | Heat shock protein 90 alpha family class A member 1 | 63601 | 5.17 | 1100 | 45 | 3.02 |
| 17 | A0A5F4CH08_CANLF | Glial fibrillary acidic protein | 54208 | 5.69 | 718 | 31.1 | 3.00 |
| 18 | E2R4E7_CANLF | Serum albumin | 67261 | 5.34 | 1116 | 41.2 | 2.82 |
| 19 | ALBU_CANLF | Albumin | 68560 | 5.52 | 2366 | 66.3 | 2.82 |
| 20 | A0A5F4BU46_CANLF | Histone H2B | 45675 | 10.93 | 299 | 16.3 | 2.77 |
| 21 | A0A5F4BWW2_CANLF | Alpha-1,4 glucan phosphorylase | 87378 | 8.62 | 417 | 25.1 | 2.75 |
| 22 | J9NYE6_CANLF | Uncharacterized protein | 25815 | 9.1 | 148 | 21.6 | 2.73 |
| 23 | F1PRZ1_CANLF | Voltage-dependent anion-selective channel protein 1 | 30722 | 8.62 | 398 | 39.2 | 2.68 |
| 24 | E2REM0_CANLF | Cytochrome c1 | 35258 | 8.93 | 189 | 28 | 2.63 |
| 25 | A0A5F4C611_CANLF | Pyruvate kinase | 64336 | 7.96 | 1269 | 42.8 | 2.61 |
| 26 | F1PPF7_CANLF | Hexokinase | 104191 | 6.07 | 947 | 31.8 | 2.60 |
| 27 | E2RFV4_CANLF | Vesicle-fusing ATPase | 91037 | 8.62 | 509 | 26 | 2.58 |
| 28 | F1PDN4_CANLF | Contactin associated protein 1 | 155541 | 6.71 | 151 | 6.3 | 2.50 |
| 29 | A0A5F4C5Z1_CANLF | Clathrin heavy chain | 180035 | 5.52 | 247 | 10.8 | 2.50 |
| 30 | A0A5F4D6L0_CANLF | Clathrin heavy chain | 191938 | 5.48 | 704 | 16.8 | 2.48 |
| 31 | A0A5F4D5V0_CANLF | Cell division control protein 42 homolog | 21215 | 6.15 | 222 | 22.5 | 2.47 |
| 32 | E2RLS3_CANLF | Heat shock protein 90 alpha family class B member 1 | 83185 | 4.97 | 1012 | 38.1 | 2.47 |
| 33 | F1PIC7_CANLF | 78 kDa glucose-regulated protein | 72238 | 5.06 | 725 | 30.4 | 2.46 |
| 34 | A0A5F4CT62_CANLF | 14-3-3 protein theta | 27747 | 4.68 | 478 | 46.9 | 2.46 |
| 35 | J9P069_CANLF | 14_3_3 domain-containing protein | 26634 | 4.67 | 317 | 24.9 | 2.37 |
| 36 | A0A5F4CN31_CANLF | Glucose-6-phosphate isomerase | 60027 | 7.44 | 562 | 26.7 | 2.35 |
| 37 | E2QW06_CANLF | ATP synthase F(0) complex subunit B1, mitochondrial | 28788 | 9.38 | 228 | 31.6 | 2.30 |
| 38 | A0A5F4BSH6_CANLF | Carbonic anhydrase | 29304 | 6.3 | 152 | 25.8 | 2.24 |
| 39 | A0A5F4C968_CANLF | Prohibitin | 35577 | 10.29 | 141 | 34.8 | 2.22 |
| 40 | A0A5F4CJ12_CANLF | Uncharacterized protein | 44794 | 7.55 | 163 | 21.7 | 2.21 |
| 41 | A0A5F4CYV2_CANLF | Succinate dehydrogenase [ubiquinone] flavoprotein subunit, mitochondrial | 80194 | 8.37 | 324 | 23.6 | 2.19 |
| 42 | RAN_CANLF | GTP-binding nuclear protein Ran | 24408 | 7.01 | 157 | 14.4 | 2.18 |
| 43 | A0A5F4CVW0_CANLF | OxoGdeHyase_C domain-containing protein | 23137 | 9.39 | 110 | 19.2 | 2.17 |
| 44 | F1PSM2_CANLF | Alpha-1,4 glucan phosphorylase | 108002 | 8.71 | 193 | 13.7 | 2.17 |
| 45 | E2R1R4_CANLF | Uncharacterized protein | 26157 | 8.44 | 152 | 32.3 | 2.15 |
| 46 | F1PMF0_CANLF | Sodium/potassium-transporting ATPase subunit beta | 34731 | 8.48 | 267 | 34.4 | 2.11 |
| 47 | A0A5F4C0I1_CANLF | Voltage-dependent anion-selective channel protein 1 | 31379 | 7.85 | 171 | 16.5 | 2.09 |
| 48 | A0A5F4CP57_CANLF | NAD(P)H quinone dehydrogenase 1 | 30437 | 8.48 | 86 | 19.2 | 2.09 |
| 49 | F1PE67_CANLF | AP-2 complex subunit alpha | 107609 | 6.63 | 199 | 14.1 | 2.08 |
| 50 | A0A5F4BYA5_CANLF | Annexin | 35738 | 10.16 | 552 | 49.1 | 2.08 |
| 51 | A0A5F4DB33_CANLF | Uncharacterized protein | 59577 | 5.26 | 284 | 13.7 | 2.07 |
| 52 | A0A5F4CRM1_CANLF | Serum albumin | 68020 | 5.69 | 1205 | 42.4 | 2.06 |
| 53 | A0A5F4CUB2_CANLF | Endoplasmin | 92621 | 4.85 | 276 | 17.6 | 2.04 |
| 54 | F1PL53_CANLF | Sodium/potassium-transporting ATPase subunit alpha | 112185 | 5.47 | 1639 | 34.7 | 2.04 |
| 55 | A0A5F4CFX8_CANLF | Alpha-1,4 glucan phosphorylase | 99317 | 7.32 | 116 | 7.9 | 2.00 |
| 56 | A0A5F4CNN8_CANLF | Anoctamin | 102352 | 7.75 | 66 | 5.2 | 2.00 |
| 57 | J9PBM4_CANLF | Ring finger and WD repeat domain 3 | 82565 | 6.48 | 62 | 10 | 2.00 |
| 58 | A0A5F4BP36_CANLF | Solute carrier family 25 member 11 | 71276 | 9.89 | 335 | 16.5 | - * |
| 59 | A0A5F4BPK6_CANLF | Dystrophin | 424499 | 5.64 | 136 | 5.8 | - * |
| 60 | A0A5F4BR02_CANLF | T-complex protein 1 subunit gamma | 56489 | 6.42 | 72 | 14.8 | - * |
| 61 | A0A5F4BR51_CANLF | Actin related protein 3 | 40731 | 5.65 | 41 | 12.1 | - * |
| 62 | A0A5F4BRB9_CANLF | Alpha-centractin | 45038 | 6.35 | 109 | 15.1 | - * |
| 63 | A0A5F4BS70_CANLF | Peptidase S1 domain-containing protein | 34360 | 5.78 | 202 | 23.3 | - * |
| 64 | A0A5F4BSM3_CANLF | 2-phospho-D-glycerate hydrolyase | 34673 | 4.83 | 651 | 52.1 | - * |
| 65 | A0A5F4BSQ8_CANLF | Karyopherin subunit beta 1 | 97351 | 4.68 | 137 | 12.3 | - * |
| 66 | A0A5F4BTN3_CANLF | Histone H2A | 14284 | 10.45 | 58 | 16.9 | - * |
| 67 | A0A5F4BTW0_CANLF | Galactokinase | 46787 | 9.31 | 89 | 13.5 | - * |
| 68 | A0A5F4BXI3_CANLF | Dynamin-1-like protein | 79329 | 8.36 | 97 | 20.3 | - * |
| 69 | A0A5F4BY90_CANLF | N-myc downstream-regulated gene 2 protein | 38666 | 5.33 | 68 | 11.4 | - * |
| 70 | A0A5F4BYJ5_CANLF | Uncharacterized protein | 50268 | 8.17 | 246 | 15.8 | - * |
| 71 | A0A5F4C2N1_CANLF | Serum albumin | 65745 | 6.3 | 39 | 10.6 | - * |
| 72 | A0A5F4C344_CANLF | Teneurin transmembrane protein 2 | 293486 | 6.31 | 62 | 5.7 | - * |
| 73 | A0A5F4C3T3_CANLF | 2-phospho-D-glycerate hydrolyase | 57403 | 7.16 | 794 | 37.1 | - * |
| 74 | A0A5F4C4P3_CANLF | Peripherin | 52485 | 6.23 | 104 | 10.6 | - * |
| 75 | A0A5F4C534_CANLF | Tropomyosin 1 | 49621 | 5.08 | 138 | 23.3 | - * |
| 76 | A0A5F4C5W5_CANLF | 14_3_3 domain-containing protein | 41580 | 9.2 | 101 | 19.9 | - * |
| 77 | A0A5F4C6G2_CANLF | Transket_pyr domain-containing protein | 90762 | 6.29 | 77 | 9.9 | - * |
| 78 | A0A5F4CFV1_CANLF | Tropomyosin 1 | 40987 | 5.06 | 166 | 31 | - * |
| 79 | A0A5F4CG77_CANLF | Voltage-dependent anion-selective channel protein 2 | 10094 | 7.66 | 79 | 56 | - * |
| 80 | A0A5F4CH61_CANLF | Electron transfer flavoprotein subunit alpha | 29903 | 8.91 | 179 | 31.3 | - * |
| 81 | A0A5F4CKN2_CANLF | RAP1B, member of RAS oncogene family | 16037 | 9.01 | 358 | 45.8 | - * |
| 82 | A0A5F4CN29_CANLF | Sodium/potassium-transporting ATPase subunit alpha | 114622 | 5.71 | 254 | 10.3 | - * |
| 83 | A0A5F4CNT2_CANLF | Mitochondrial carrier 2 | 33408 | 8.59 | 50 | 10.9 | - * |
| 84 | A0A5F4CQ53_CANLF | Complex I subunit B13 | 13442 | 5.9 | 85 | 22.2 | - * |
| 85 | A0A5F4CRD6_CANLF | Histone cluster 1 H1 family member b | 22747 | 10.97 | 94 | 36.7 | - * |
| 86 | A0A5F4CSG0_CANLF | Dihydrolipoyl dehydrogenase, mitochondrial | 49270 | 7.19 | 115 | 16.3 | - * |
| 87 | A0A5F4CXU8_CANLF | Uncharacterized protein | 25342 | 8.27 | 57 | 9.9 | - * |
| 88 | A0A5F4D092_CANLF | Flavin-containing monooxygenase | 32555 | 8.66 | 42 | 7.7 | - * |
| 89 | A0A5F4D116_CANLF | Myelin associated oligodendrocyte basic protein | 18154 | 11.01 | 65 | 32.5 | - * |
| 90 | A0A5F4D1M0_CANLF | Peripherin | 51371 | 5.31 | 96 | 11 | - * |
| 91 | A0A5F4D2Q5_CANLF | HECT and RLD domain containing E3 ubiquitin protein ligase 4 | 106551 | 6.04 | 58 | 5.8 | - * |
| 92 | A0A5F4D598_CANLF | Ubiquitin carboxyl-terminal hydrolase | 38049 | 8.92 | 346 | 33 | - * |
| 93 | A0A5F4D5F7_CANLF | Serpin family A member 1 | 44944 | 6.06 | 126 | 7 | - * |
| 94 | A0A5F4D5Y0_CANLF | Ubiquitin conjugating enzyme E2 E3 | 16914 | 5.75 | 44 | 21.1 | - * |
| 95 | A0A5F4D804_CANLF | Voltage-dependent anion-selective channel protein 2 | 31565 | 6.72 | 308 | 33.3 | - * |
| 96 | A0A5F4D992_CANLF | Dynein axonemal heavy chain 1 | 494159 | 5.72 | 124 | 7.2 | - * |
| 97 | A0A5F4DA37_CANLF | Quinoid dihydropteridine reductase | 22220 | 9.23 | 113 | 55.5 | - * |
| 98 | A0A5F4DCE0_CANLF | Tropomyosin 1 | 39196 | 6.36 | 220 | 32.8 | - * |
| 99 | A0A5F4DCJ4_CANLF | Vacuolar protein sorting-associated protein 35 | 85995 | 5.23 | 54 | 3.1 | - * |
| 100 | A0A5F4DDK9_CANLF | Purine nucleoside phosphorylase | 32238 | 6.4 | 108 | 13.5 | - * |
| 101 | A0A5F4DEC1_CANLF | Uncharacterized protein | 50088 | 8.7 | 81 | 12.9 | - * |
| 102 | A0A5F4DFY2_CANLF | Glutaminase | 78572 | 9.54 | 88 | 11.8 | - * |
| 103 | A0A5F4DHW3_CANLF | Programmed cell death 6 interacting protein | 85974 | 6.18 | 47 | 8.4 | - * |
| 104 | A0A5F4DI49_CANLF | Diadenosine tetraphosphate synthetase | 89943 | 9.01 | 78 | 9.4 | - * |
| 105 | E2QUA8_CANLF | Cytochrome c oxidase subunit 4I1 | 19563 | 9.21 | 64 | 16 | - * |
| 106 | E2R494_CANLF | H15 domain-containing protein | 30979 | 10.63 | 74 | 16 | - * |
| 107 | E2R578_CANLF | Solute carrier family 25 member 11 | 34054 | 9.95 | 352 | 31.8 | - * |
| 108 | E2R9F5_CANLF | Hydroxysteroid 17-beta dehydrogenase 10 | 27138 | 8.44 | 291 | 45.2 | - * |
| 109 | E2R9N5_CANLF | RNA binding motif protein 12 | 59807 | 5.64 | 51 | 3.7 | - * |
| 110 | E2RAD2_CANLF | Coiled-coil domain-containing protein 181 | 59951 | 5.44 | 60 | 3.5 | - * |
| 111 | E2RFW7_CANLF | Oligodendrocyte myelin glycoprotein | 49497 | 8.56 | 51 | 4.1 | - * |
| 112 | E2RKR4_CANLF | Proteasome subunit alpha type | 27369 | 6.96 | 57 | 17.9 | - * |
| 113 | E2RNB6_CANLF | Alpha(B)-crystallin | 20054 | 6.76 | 459 | 52 | - * |
| 114 | E2RRD2_CANLF | H15 domain-containing protein | 20916 | 11.02 | 88 | 23.6 | - * |
| 115 | E2RS09_CANLF | Glial fibrillary acidic protein | 49987 | 5.36 | 516 | 42.3 | - * |
| 116 | E2RT63_CANLF | Heat shock 70 kDa protein 4 | 88455 | 5.06 | 132 | 12.5 | - * |
| 117 | F1P767_CANLF | Sodium/potassium-transporting ATPase subunit alpha | 113015 | 5.27 | 1639 | 35.9 | - * |
| 118 | F1PAR8_CANLF | Prohibitin | 28850 | 5.76 | 171 | 27.9 | - * |
| 119 | F1PBJ8_CANLF | MAPK activated protein kinase 5 | 108534 | 7.3 | 75 | 9.6 | - * |
| 120 | F1PGM1_CANLF | C3-beta-c | 176028 | 6.74 | 60 | 6.8 | - * |
| 121 | F1PI25_CANLF | Sidoreflexin | 35691 | 9.2 | 364 | 36.6 | - * |
| 122 | F1PJS0_CANLF | Glyoxylate and hydroxypyruvate reductase | 35632 | 7.59 | 52 | 12.8 | - * |
| 123 | F1PR71_CANLF | Syntaxin 2 | 31951 | 5.42 | 48 | 16.9 | - * |
| 124 | F1PTX2_CANLF | NAD-dependent protein deacetylase | 35558 | 7.34 | 122 | 28.3 | - * |
| 125 | F1PU03_CANLF | Amino acid transporter | 62681 | 5.99 | 331 | 24.2 | - * |
| 126 | F1PUA2_CANLF | Acetyl-coenzyme A synthetase | 93781 | 9.44 | 59 | 6.6 | - * |
| 127 | F1PVF1_CANLF | Uncharacterized protein | 25808 | 9.52 | 83 | 26.1 | - * |
| 128 | F1PX75_CANLF | Protein phosphatase 2 scaffold subunit Aalpha | 65281 | 5 | 66 | 19.7 | - * |
| 129 | F1Q231_CANLF | Reticulon | 76143 | 4.55 | 72 | 10.6 | - * |
| 130 | F2Z4N8_CANLF | Deoxyguanosine kinase | 41850 | 5.31 | 864 | 34.3 | - * |
| 131 | F6UWI0_CANLF | Ribosomal protein S6 kinase | 85530 | 8.99 | 57 | 13.5 | - * |
| 132 | F6X6E5_CANLF | Collapsin response mediator protein 1 | 74202 | 6.36 | 172 | 15.3 | - * |
| 133 | F6XRY2_CANLF | Eukaryotic translation elongation factor 2 | 95279 | 6.41 | 164 | 12 | - * |
| 134 | G1K296_CANLF | ATP-dependent 6-phosphofructokinase | 93660 | 8.2 | 145 | 11.4 | - * |
| 135 | J9JHP2_CANLF | Histone H2A | 22067 | 11.7 | 165 | 19.6 | - * |
| 136 | J9NSF3_CANLF | Voltage-dependent anion-selective channel protein 3 | 30749 | 8.95 | 187 | 22.5 | - * |
| 137 | J9NSU7_CANLF | Dimethylargininase | 31325 | 5.95 | 120 | 26 | - * |
| 138 | J9NT18_CANLF | L-lactate dehydrogenase | 36532 | 5.48 | 208 | 20.1 | - * |
| 139 | J9NT20_CANLF | Obg-like ATPase 1 | 47149 | 8.4 | 77 | 15.2 | - * |
| 140 | J9NTP4_CANLF | Multifunctional fusion protein | 17440 | 6.21 | 69 | 29.7 | - * |
| 141 | J9NTY1_CANLF | RAB39B, member RAS oncogene family | 23992 | 7.94 | 75 | 10.5 | - * |
| 142 | J9NUR6_CANLF | ATP synthase subunit d, mitochondrial | 19761 | 5.14 | 255 | 43.3 | - * |
| 143 | J9NWA7_CANLF | Ras-related protein Rab-2A | 22027 | 6.08 | 85 | 33.2 | - * |
| 144 | J9NWP4_CANLF | Dedicator of cytokinesis 7 | 230342 | 6.56 | 86 | 10.8 | - * |
| 145 | J9NYE0_CANLF | COP9 signalosome complex subunit 8 | 23199 | 5.25 | 56 | 12 | - * |
| 146 | J9P114_CANLF | ATP synthase subunit d, mitochondrial | 18663 | 5.26 | 172 | 35.4 | - * |
| 147 | J9P371_CANLF | Histone H3 | 15292 | 10.7 | 83 | 24.3 | - * |
| 148 | J9P550_CANLF | Uncharacterized protein | 21228 | 5.16 | 142 | 30.2 | - * |
| 149 | J9P7X1_CANLF | Histone H3 | 15379 | 11.27 | 99 | 33.8 | - * |
| 150 | L7N071_CANLF | Actinin alpha 4 | 131647 | 5.95 | 160 | 11.3 | - * |
| 151 | RAB7A_CANLF | Ras-related protein Rab-7a | 23505 | 6.4 | 97 | 26.6 | - * |
| 152 | A0A5F4C3D3_CANLF | Synaptosome associated protein 91 | 83555 | 5.13 | 112 | 4.9 | -2.00 |
| 153 | A0A5F4C9W9_CANLF | Biliverdin reductase B | 26164 | 6.89 | 111 | 18.8 | -2.08 |
| 154 | F1PNI3_CANLF | Fructose-bisphosphate aldolase | 36704 | 8.36 | 351 | 31.5 | -2.08 |
| 155 | J9P5E0_CANLF | Pyridoxal kinase | 30144 | 6.15 | 85 | 18.4 | -2.09 |
| 156 | F1PFN3_CANLF | Glyceraldehyde-3-phosphate dehydrogenase | 40104 | 6.5 | 409 | 35.9 | -2.11 |
| 157 | A0A5F4C1F7_CANLF | Uncharacterized protein | 47061 | 8.3 | 88 | 10.4 | -2.14 |
| 158 | A0A5F4DI44_CANLF | Gp_dh_N domain-containing protein | 30207 | 6.5 | 142 | 33.9 | -2.17 |
| 159 | F1P6Q0_CANLF | G protein subunit alpha i3 | 40536 | 5.5 | 130 | 31.4 | -2.17 |
| 160 | A0A5F4DA52_CANLF | Gp_dh_N domain-containing protein | 35562 | 6.05 | 490 | 58.6 | -2.20 |
| 161 | F1PVP1_CANLF | Syntaxin 1A | 35178 | 5.02 | 100 | 20.8 | -2.22 |
| 162 | J9NWZ6_CANLF | Glyceraldehyde-3-phosphate dehydrogenase | 36185 | 7.21 | 322 | 34.2 | -2.23 |
| 163 | A0A5F4D839_CANLF | Gp_dh_N domain-containing protein | 30283 | 6.75 | 464 | 47.9 | -2.50 |
| 164 | E2RIQ8_CANLF | Ribosomal protein S5 | 22862 | 9.73 | 206 | 19.1 | -2.54 |
| 165 | E2RHM4_CANLF | Isocitrate dehydrogenase [NAD] subunit, mitochondrial | 39732 | 6.27 | 161 | 18.6 | -2.58 |
| 166 | E2R907_CANLF | Synaptosomal-associated protein | 23321 | 4.74 | 243 | 57.3 | -2.61 |
| 167 | F1PMS8_CANLF | Uncharacterized protein | 122422 | 4.79 | 440 | 14.8 | -2.69 |
| 168 | A0A5F4BSA6_CANLF | G protein subunit alpha o1 | 40047 | 5.62 | 594 | 35.9 | -2.70 |
| 169 | F1PVS8_CANLF | V-type proton ATPase subunit a | 92850 | 6.53 | 182 | 13.4 | -2.73 |
| 170 | A0A5F4CDP2_CANLF | Uncharacterized protein | 18794 | 4.84 | 132 | 22.4 | -2.86 |
| 171 | F1PQT9_CANLF | Synapsin I | 70151 | 9.84 | 251 | 16 | -2.98 |
| 172 | J9NTB4_CANLF | Uncharacterized protein | 29385 | 8.89 | 140 | 12.5 | -3.03 |
| 173 | F1PYU9_CANLF | Keratin, type I cytoskeletal 10 | 57650 | 5.09 | 264 | 13.4 | -3.06 |
| 174 | E2RJQ8_CANLF | V-type proton ATPase subunit | 40375 | 4.85 | 190 | 13.1 | -3.38 |
| 175 | E2RPM2_CANLF | Synaptosomal-associated protein | 23300 | 4.66 | 253 | 48.1 | -3.55 |
| 176 | J9P540_CANLF | Glyceraldehyde-3-phosphate dehydrogenase | 36141 | 7.16 | 308 | 35 | -3.60 |
| 177 | K2C1_CANLF | Keratin, type II cytoskeletal 1 | 63751 | 7.66 | 203 | 17.4 | -3.73 |
| 178 | J9P7B8_CANLF | Synaptobrevin-2 | 12641 | 7.85 | 174 | 35.3 | -3.89 |
| 179 | A0A5F4DB38_CANLF | Peptidase S1 domain-containing protein | 35574 | 5.87 | 418 | 41.6 | -4.16 |
| 180 | A0A5F4CSY2_CANLF | Apolipoprotein E | 36582 | 5.41 | 461 | 34.2 | -4.18 |
| 181 | J9P8P9_CANLF | Calcium-transporting ATPase | 133114 | 5.57 | 366 | 14.7 | -4.33 |
| 182 | A0A5F4BNL7_CANLF | Uncharacterized protein | 41964 | 9.13 | 40 | 13.8 | 0.00 |
| 183 | A0A5F4BNZ8_CANLF | V-type proton ATPase subunit a | 91812 | 7.55 | 187 | 11.6 | 0.00 |
| 184 | A0A5F4BPT0_CANLF | Ras-related protein Rab-7a | 25689 | 6.43 | 141 | 21.9 | 0.00 |
| 185 | A0A5F4BR59_CANLF | Gp_dh_N domain-containing protein | 18039 | 6.89 | 106 | 25.2 | 0.00 |
| 186 | A0A5F4BX60_CANLF | Sodium/potassium-transporting ATPase subunit alpha | 115197 | 5.62 | 1059 | 36.3 | 0.00 |
| 187 | A0A5F4BY58_CANLF | Syntaxin 2 | 34422 | 5.28 | 50 | 6 | 0.00 |
| 188 | A0A5F4C3S4_CANLF | Uncharacterized protein | 45251 | 6.1 | 122 | 11.7 | 0.00 |
| 189 | A0A5F4C3X9_CANLF | Actinin alpha 4 | 131646 | 5.95 | 131 | 11.8 | 0.00 |
| 190 | A0A5F4C5K5_CANLF | RAP1B, member of RAS oncogene family | 18781 | 8.72 | 327 | 30.3 | 0.00 |
| 191 | A0A5F4C7H4_CANLF | Succinate dehydrogenase [ubiquinone] flavoprotein subunit, mitochondrial | 67528 | 8.52 | 210 | 22.4 | 0.00 |
| 192 | A0A5F4C8K4_CANLF | Peptidyl-prolyl cis-trans isomerase | 13992 | 5.73 | 55 | 26 | 0.00 |
| 193 | A0A5F4C8V1_CANLF | F-actin-capping protein subunit beta | 41837 | 5.33 | 101 | 16.5 | 0.00 |
| 194 | A0A5F4CA54_CANLF | 1-phosphatidylinositol 4,5-bisphosphate phosphodiesterase | 132738 | 5.83 | 74 | 8.4 | 0.00 |
| 195 | A0A5F4CEA4_CANLF | RAB37, member RAS oncogene family | 22221 | 6.32 | 67 | 41.8 | 0.00 |
| 196 | A0A5F4CF09_CANLF | Actin alpha cardiac muscle 1 | 49918 | 5.75 | 724 | 32 | 0.00 |
| 197 | A0A5F4CGS5_CANLF | Fascin actin-bundling protein 1 | 84854 | 8.71 | 103 | 7.3 | 0.00 |
| 198 | A0A5F4CJ11_CANLF | Bactericidal permeability-increasing protein | 64702 | 9.46 | 51 | 14.2 | 0.00 |
| 199 | A0A5F4CP83_CANLF | Amino acid transporter | 62013 | 6.4 | 328 | 36.3 | 0.00 |
| 200 | A0A5F4CPQ6_CANLF | CCT-theta | 77059 | 8.09 | 61 | 14.2 | 0.00 |
| 201 | A0A5F4CQ93_CANLF | Vacuolar protein sorting-associated protein 18 homolog | 113729 | 5.94 | 40 | 10.2 | 0.00 |
| 202 | A0A5F4CUU0_CANLF | Doublecortin like kinase 2 | 82147 | 8.68 | 47 | 9.8 | 0.00 |
| 203 | A0A5F4CVM7_CANLF | Ubiquitin specific peptidase 8 | 114978 | 8.52 | 55 | 10.6 | 0.00 |
| 204 | A0A5F4CZL0_CANLF | Glial fibrillary acidic protein | 56336 | 6.29 | 688 | 30.3 | 0.00 |
| 205 | A0A5F4D1K3_CANLF | PKS_ER domain-containing protein | 31873 | 9.03 | 82 | 12.2 | 0.00 |
| 206 | A0A5F4D6S6_CANLF | Vacuolar protein sorting-associated protein 29 | 20483 | 6.29 | 49 | 7.1 | 0.00 |
| 207 | A0A5F4D8M4_CANLF | Cofilin, non-muscle isoform | 26406 | 8.17 | 66 | 17.2 | 0.00 |
| 208 | A0A5F4D8X1_CANLF | High density lipoprotein binding protein | 171182 | 9.55 | 53 | 7.3 | 0.00 |
| 209 | A0A5F4DAD7_CANLF | IF rod domain-containing protein | 49973 | 4.91 | 56 | 14.3 | 0.00 |
| 210 | A0A5F4DB56_CANLF | Multidrug and toxin extrusion protein | 59457 | 9.14 | 62 | 9.2 | 0.00 |
| 211 | A0A5F4DDG2_CANLF | Myristoylated alanine rich protein kinase C substrate | 33805 | 4.38 | 55 | 4.5 | 0.00 |
| 212 | A0A5F4DGP5_CANLF | Doublecortin like kinase 2 | 81669 | 8.89 | 48 | 17.2 | 0.00 |
| 213 | A0A5F4DHK7_CANLF | 116 kDa U5 small nuclear ribonucleoprotein component | 109380 | 4.85 | 57 | 9.3 | 0.00 |
| 214 | A0A5F4DK65_CANLF | 1-phosphatidylinositol 4,5-bisphosphate phosphodiesterase | 135802 | 6.37 | 65 | 10.3 | 0.00 |
| 215 | E2QTQ9_CANLF | Tubulin alpha chain | 49180 | 4.96 | 1154 | 50 | 0.00 |
| 216 | E2R0U2_CANLF | Glycine-N-acyltransferase like 3 | 32186 | 9 | 66 | 22.2 | 0.00 |
| 217 | E2R150_CANLF | Keratin 24 | 55901 | 4.83 | 86 | 7.9 | 0.00 |
| 218 | E2R1Z3_CANLF | IF rod domain-containing protein | 50453 | 5.27 | 44 | 3.9 | 0.00 |
| 219 | E2R261_CANLF | IF rod domain-containing protein | 48538 | 4.29 | 67 | 8.1 | 0.00 |
| 220 | E2R280_CANLF | Nebulin | 668371 | 9.15 | 130 | 8.1 | 0.00 |
| 221 | E2R2N2_CANLF | Mitogen-activated protein kinase | 41363 | 6.5 | 102 | 24.7 | 0.00 |
| 222 | E2R3Y6_CANLF | IF rod domain-containing protein | 46326 | 4.83 | 85 | 13.2 | 0.00 |
| 223 | E2R708_CANLF | IF rod domain-containing protein | 50795 | 4.91 | 64 | 14.9 | 0.00 |
| 224 | E2R7T3_CANLF | Keratin 35 | 50325 | 4.96 | 52 | 7 | 0.00 |
| 225 | E2R8Q7_CANLF | IF rod domain-containing protein | 56085 | 5.61 | 95 | 10 | 0.00 |
| 226 | E2R948_CANLF | Voltage-dependent anion-selective channel protein 2 | 34477 | 8.45 | 166 | 15 | 0.00 |
| 227 | E2RAV7_CANLF | Fat storage inducing transmembrane protein 2 | 43598 | 10.14 | 45 | 4.9 | 0.00 |
| 228 | E2RBL4_CANLF | FXYD domain-containing ion transport regulator | 11960 | 4.91 | 65 | 23.4 | 0.00 |
| 229 | E2RC93_CANLF | Ras-related protein Rab-3 | 25898 | 5.09 | 165 | 30.4 | 0.00 |
| 230 | E2RCL5_CANLF | Ketimine reductase mu-crystallin | 33661 | 5.07 | 88 | 15.6 | 0.00 |
| 231 | E2REU6_CANLF | IF rod domain-containing protein | 48209 | 5.33 | 58 | 3.7 | 0.00 |
| 232 | E2RG57_CANLF | Calcium/calmodulin-dependent protein kinase | 59104 | 7.06 | 209 | 19.9 | 0.00 |
| 233 | E2RGI7_CANLF | Arginine and glutamate rich 1 | 37736 | 7.68 | 45 | 12.9 | 0.00 |
| 234 | E2RK38_CANLF | Tr-type G domain-containing protein | 51940 | 9.13 | 71 | 10.3 | 0.00 |
| 235 | E2RPP4_CANLF | Non-specific serine/threonine protein kinase | 191242 | 6.18 | 69 | 7.1 | 0.00 |
| 236 | F1P669_CANLF | Synapsin II | 59374 | 7.83 | 49 | 13.8 | 0.00 |
| 237 | F1P7C2_CANLF | Alanine--tRNA ligase | 106709 | 5.27 | 80 | 12.8 | 0.00 |
| 238 | F1PC59_CANLF | Peroxiredoxin-6 | 24884 | 5.71 | 122 | 29.1 | 0.00 |
| 239 | F1PG13_CANLF | Ubiquitin conjugating enzyme E2 O | 140882 | 5.05 | 68 | 10.5 | 0.00 |
| 240 | F1PHH6_CANLF | Guanine deaminase | 55190 | 5.96 | 144 | 20.4 | 0.00 |
| 241 | F1PK01_CANLF | Dihydropyrimidinase like 5 | 61426 | 6.73 | 51 | 18.8 | 0.00 |
| 242 | F1PW89_CANLF | Phenylethanolamine N-methyltransferase | 48155 | 8.91 | 51 | 5.5 | 0.00 |
| 243 | F1PYE8_CANLF | Gp_dh_C domain-containing protein | 16848 | 9.42 | 213 | 43.3 | 0.00 |
| 244 | F1Q088_CANLF | Dihydropyrimidinase like 4 | 71224 | 8.43 | 69 | 11.6 | 0.00 |
| 245 | F1Q0N9_CANLF | IF rod domain-containing protein | 57608 | 5.81 | 97 | 13.4 | 0.00 |
| 246 | F6Y4R5_CANLF | Patatin like phospholipase domain containing 8 | 88247 | 9.36 | 69 | 9.3 | 0.00 |
| 247 | J9JHZ0_CANLF | Ldh_1_N domain-containing protein | 21241 | 7.6 | 127 | 20.6 | 0.00 |
| 248 | J9NU18_CANLF | ATP binding cassette subfamily A member 5 | 186872 | 6.64 | 51 | 2.5 | 0.00 |
| 249 | J9NX28_CANLF | Small monomeric GTPase | 23525 | 6.66 | 69 | 6.8 | 0.00 |
| 250 | J9NXS3_CANLF | Peptidyl-prolyl cis-trans isomerase | 15745 | 8.95 | 97 | 33.8 | 0.00 |
| 251 | J9P3Y9_CANLF | Nebulin | 756061 | 9.11 | 155 | 8.3 | 0.00 |
| 252 | J9P4H9_CANLF | IF rod domain-containing protein | 48655 | 4.82 | 113 | 15.3 | 0.00 |
| 253 | J9P5I6_CANLF | Immunoglobulin superfamily member 8 | 66627 | 8.42 | 93 | 17 | 0.00 |
| 254 | L7N0G8_CANLF | Keratin 36 | 51990 | 4.97 | 59 | 10.5 | 0.00 |
| 255 | L7N0G9_CANLF | Keratin 32 | 50520 | 4.84 | 72 | 24 | 0.00 |

* means proteins only found in RABV-positive brains
