# Supplementary material for: Proteomic and phosphoproteomic analysis of rabies pathogenesis in the clinical canine brain and identification of a kinase inhibitor as a potential repurposed antiviral agent
Source: PLoS One. 2025 Jun 27;20(6):e0323931. doi: 10.1371/journal.pone.0323931 (PMC12204518; doi:10.1371/journal.pone.0323931)
Supplement: S2 Table — (DOCX) [file pone.0323931.s004.docx]

**Table S2. Gene ontology analysis of proteomic and phosphoproteomic data**

| **Gene ontology category** | | **Proteomic data** | | **Phosphoproteomic data** | |
| --- | --- | --- | --- | --- | --- |
|  |  | **Protein number** | **%** | **Protein number** | **%** |
| Molecular function | Binding | 39 | 23.8 | 3 | 27.3 |
|  | Catalytic activity | 36 | 22 | 6 | 54.5 |
|  | Transporter activity | 9 | 5.5 | 0 | 0 |
|  | ATP-dependent activity | 6 | 3.7 | 1 | 9.1 |
|  | Molecular adaptor activity | 4 | 2.4 | 0 | 0 |
|  | Molecular function regulator | 3 | 1.8 | 0 | 0 |
|  | Structural molecule activity | 3 | 1.8 | 0 | 0 |
|  | Translation regulator activity | 2 | 1.2 | 0 | 0 |
|  | Molecular transducer activity | 1 | 0.6 | 0 | 0 |
|  | Unassigned category | 61 | 37.2 | 1 | 9.1 |
| Biological process | Cellular process | 70 | 27.6 | 7 | 41.2 |
|  | Metabolic process | 29 | 11.4 | 3 | 17.6 |
|  | Localization | 28 | 11 | 2 | 11.8 |
|  | Biological regulation | 27 | 10.6 | 1 | 5.9 |
|  | Signaling | 13 | 5.1 | 1 | 5.9 |
|  | Response to stimulus | 12 | 4.7 | 2 | 11.8 |
|  | Developmental process | 7 | 2.8 | 0 | 0 |
|  | Multicellular organismal process | 7 | 2.8 | 0 | 0 |
|  | Locomotion | 3 | 1.2 | 0 | 0 |
|  | Unassigned category | 58 | 22.8 | 1 | 5.9 |
| Cellular component | Cellular anatomical entity | 78 | 53.4 | 5 | 55.6 |
|  | Protein-containing complex | 17 | 11.6 | 1 | 11.1 |
|  | Unassigned category | 51 | 34.9 | 3 | 33.3 |
