# Supplementary material for: Proteomic and phosphoproteomic analysis of rabies pathogenesis in the clinical canine brain and identification of a kinase inhibitor as a potential repurposed antiviral agent
Source: PLoS One. 2025 Jun 27;20(6):e0323931. doi: 10.1371/journal.pone.0323931 (PMC12204518; doi:10.1371/journal.pone.0323931)
Supplement: S3 Table — (DOCX) [file pone.0323931.s005.docx]

**Table S3 Differentially phosphorylated proteins from RABV-positive dog brains, in comparison with RABV-negative brains.**

| **No.** | **Accession No.** | **Protein Name** | **MW** | **pI** | **Protein Score** | **Sequence Coverage** | **Average Fold-change** |
| --- | --- | --- | --- | --- | --- | --- | --- |
| 1 | KCRB_CANLF | Creatine phosphokinase M-type | 42674 | 5.47 | 59 | 2.9 | 5.53 |
| 2 | XP_005619707.1 | Heat shock cognate 71 kDa protein | 70854 | 5.37 | 93 | 4.5 | 5.00 |
| 3 | XP_849125.1 | Dihydropyrimidinase-related protein 2 isoform X1 | 73545 | 5.98 | 99 | 8 | 4.52 |
| 4 | XP_022262693.1 | Peroxiredoxin-2 isoform X1 | 26016 | 5.69 | 72 | 20.1 | 2.08 |
| 5 | RCN39677.1 | Hypothetical protein ANCCAN_14376 | 70387 | 5.36 | 57 | 4.5 | 2.00 |
| 6 | XP_038474787.1 | Syntaxin-binding protein 1 isoform X1 | 68692 | 6.32 | 121 | 8.5 | 2.00 |
| 7 | ANW72321.1 | Mitochondrial ATP synthase subunit alpha subunit | 59691 | 9.22 | 156 | 11.2 | - * |
| 8 | ANW72322.1 | Mitochondrial ATP synthase subunit beta subunit | 56250 | 5.21 | 111 | 15.6 | - * |
| 9 | AQX24407.1 | Molecular chaperone DnaK | 68202 | 4.85 | 94 | 9.2 | - * |
| 10 | AUZ82886.1 | Immunoglobulin heavy chain variable region, partial | 13625 | 4.96 | 75 | 32.3 | - * |
| 11 | AUZ82902.1 | Immunoglobulin heavy chain variable region, partial | 13207 | 6.45 | 68 | 33.3 | - * |
| 12 | NP_001332968.1 | Keratin, type II cytoskeletal 8 | 54684 | 5.66 | 66 | 5.3 | - * |
| 13 | pdb\|2QLS\|B | Chain B, Hemoglobin subunit beta | 15986 | 7.96 | 175 | 31.5 | - * |
| 14 | prf\|\|1908227A | Beta spectrin | 81139 | 5.47 | 232 | 19 | - * |
| 15 | RCN42839.1 | Actin | 28193 | 5.2 | 120 | 19.4 | - * |
| 16 | RCN45414.1 | GTP-binding protein LepA | 72316 | 8.69 | 53 | 1.2 | - * |
| 17 | RCN45771.1 | Beta-ketoacyl synthase protein | 657416 | 8.44 | 102 | 3 | - * |
| 18 | RCN46266.1 | Hypothetical protein ANCCAN_07726 | 216844 | 6.61 | 68 | 1.7 | - * |
| 19 | RCN48189.1 | Actin | 42006 | 5.36 | 119 | 14.1 | - * |
| 20 | RCN48777.1 | DnaJ domain protein | 145695 | 8.91 | 50 | 2.5 | - * |
| 21 | GLNA_CANLF | Glutamate--ammonia ligase | 42001 | 6.28 | 53 | 9.7 | - * |
| 22 | XP_005625117.1 | Tubulin beta-4B chain isoform X2 | 49799 | 4.79 | 97 | 9.2 | - * |
| 23 | XP_005640555.1 | 60 kDa heat shock protein, mitochondrial | 60957 | 5.78 | 54 | 11 | - * |
| 24 | XP_038283425.1 | Transketolase | 68080 | 6.97 | 161 | 12.2 | - * |
| 25 | XP_038291098.1 | Serine/threonine-protein kinase Nek1 isoform X5 | 131462 | 5.21 | 60 | 4.4 | - * |
| 26 | XP_038297815.1 | Actin, alpha cardiac muscle 1 | 41992 | 5.23 | 210 | 23.9 | - * |
| 27 | XP_038301488.1 | Tubulin polymerization-promoting protein | 23823 | 9.47 | 64 | 16.7 | - * |
| 28 | XP_038305255.1 | Synaptotagmin-1 isoform X1 | 47583 | 8.26 | 71 | 5.9 | - * |
| 29 | XP_038308507.1 | Peptidyl-prolyl cis-trans isomerase A isoform X1 | 17858 | 8.34 | 88 | 25.6 | - * |
| 30 | XP_038316069.1 | Keratin, type II cytoskeletal 1b isoform X1 | 63007 | 7.14 | 98 | 24 | - * |
| 31 | XP_535365.2 | Heterogeneous nuclear ribonucleoprotein R isoform X4 | 59645 | 9.2 | 63 | 3.2 | - * |
| 32 | XP_536313.2 | Betaine--homocysteine S-methyltransferase 1 isoform X1 | 45074 | 7.14 | 66 | 4.9 | - * |
| 33 | XP_849434.1 | Fructose-bisphosphate aldolase A | 39478 | 8.3 | 73 | 14.8 | - * |
| 34 | XP_851125.1 | Guanine nucleotide-binding protein G(o) subunit alpha isoform X2 | 40047 | 5.62 | 121 | 15 | - * |
| 35 | BAR79439.1 | Annexin A5 | 35921 | 4.99 | 107 | 8.1 | -2.00 |
| 36 | XP_005626165.1 | Spectrin beta chain, non-erythrocytic 1 isoform X2 | 272632 | 5.44 | 541 | 5.4 | -2.25 |
| 37 | AAA30879.1 | Beta-spectrin, partial | 81069 | 5.47 | 143 | 4.8 | -3.25 |
| 38 | AUZ82936.1 | Immunoglobulin heavy chain variable region, partial | 13643 | 4.98 | 86 | 36.8 | 0.00 |
| 39 | CAD10571.1 | Unnamed protein product | 51926 | 8.52 | 258 | 12.7 | 0.00 |
| 40 | NP_001332949.1 | Keratin, type II cytoskeletal 2 oral | 66237 | 8.78 | 122 | 16.5 | 0.00 |
| 41 | RCN29942.1 | Hypothetical protein ANCCAN_24286, partial | 103913 | 5.81 | 48 | 5.2 | 0.00 |

* means proteins only found in RABV-positive brains
