# Supplementary material for: Proteomic and phosphoproteomic analysis of rabies pathogenesis in the clinical canine brain and identification of a kinase inhibitor as a potential repurposed antiviral agent
Source: PLoS One. 2025 Jun 27;20(6):e0323931. doi: 10.1371/journal.pone.0323931 (PMC12204518; doi:10.1371/journal.pone.0323931)
Supplement: S4 Table — (DOCX) [file pone.0323931.s006.docx]

**Table S4 Pathway analysis of differentially expressed proteins according to KEGG pathway.**

| **No.** | **Pathway** | **Description** | **Count in network** | **Strength** | **False discovery rate** |
| --- | --- | --- | --- | --- | --- |
| 1 | [cfa00020](https://www.kegg.jp/kegg-bin/show_pathway?cfa00020) | Citrate cycle (TCA cycle) | 4 of 27 | 1.28 | 0.0033 |
| 2 | [cfa00010](https://www.kegg.jp/kegg-bin/show_pathway?cfa00010) | Glycolysis / Gluconeogenesis | 9 of 62 | 1.27 | 1.19e^-06^ |
| 3 | [cfa00620](https://www.kegg.jp/kegg-bin/show_pathway?cfa00620) | Pyruvate metabolism | 5 of 37 | 1.24 | 0.0007 |
| 4 | [cfa04721](https://www.kegg.jp/kegg-bin/show_pathway?cfa04721) | Synaptic vesicle cycle | 8 of 65 | 1.2 | 7.27e^-06^ |
| 5 | [cfa00052](https://www.kegg.jp/kegg-bin/show_pathway?cfa00052) | Galactose metabolism | 3 of 25 | 1.19 | 0.0245 |
| 6 | [cfa00030](https://www.kegg.jp/kegg-bin/show_pathway?cfa00030) | Pentose phosphate pathway | 3 of 26 | 1.17 | 0.0245 |
| 7 | [cfa00630](https://www.kegg.jp/kegg-bin/show_pathway?cfa00630) | Glyoxylate and dicarboxylate metabolism | 3 of 27 | 1.16 | 0.0256 |
| 8 | [cfa00051](https://www.kegg.jp/kegg-bin/show_pathway?cfa00051) | Fructose and mannose metabolism | 3 of 29 | 1.13 | 0.0281 |
| 9 | [cfa00500](https://www.kegg.jp/kegg-bin/show_pathway?cfa00500) | Starch and sucrose metabolism | 3 of 30 | 1.11 | 0.0282 |
| 10 | [cfa00640](https://www.kegg.jp/kegg-bin/show_pathway?cfa00640) | Propanoate metabolism | 3 of 32 | 1.08 | 0.0317 |
| 11 | [cfa04915](https://www.kegg.jp/kegg-bin/show_pathway?cfa04915) | Estrogen signaling pathway | 10 of 110 | 1.07 | 3.39e^-06^ |
| 12 | [cfa05150](https://www.kegg.jp/kegg-bin/show_pathway?cfa05150) | *Staphylococcus aureus* infection | 5 of 57 | 1.06 | 0.0037 |
| 13 | [cfa04961](https://www.kegg.jp/kegg-bin/show_pathway?cfa04961) | Endocrine and other factor-regulated calcium reabsorption | 4 of 48 | 1.03 | 0.016 |
| 14 | [cfa01200](https://www.kegg.jp/kegg-bin/show_pathway?cfa01200) | Carbon metabolism | 9 of 112 | 1.02 | 2.61e^-05^ |
| 15 | [cfa05230](https://www.kegg.jp/kegg-bin/show_pathway?cfa05230) | Central carbon metabolism in cancer | 5 of 63 | 1.01 | 0.0053 |
| 16 | [cfa04612](https://www.kegg.jp/kegg-bin/show_pathway?cfa04612) | Antigen processing and presentation | 3 of 40 | 0.99 | 0.0482 |
| 17 | [cfa05322](https://www.kegg.jp/kegg-bin/show_pathway?cfa05322) | Systemic lupus erythematosus | 4 of 71 | 0.86 | 0.0317 |
| 18 | [cfa04670](https://www.kegg.jp/kegg-bin/show_pathway?cfa04670) | Leukocyte transendothelial migration | 5 of 97 | 0.82 | 0.0236 |
| 19 | [cfa04261](https://www.kegg.jp/kegg-bin/show_pathway?cfa04261) | Adrenergic signaling in cardiomyocytes | 6 of 126 | 0.79 | 0.0139 |
| 20 | [cfa04071](https://www.kegg.jp/kegg-bin/show_pathway?cfa04071) | Sphingolipid signaling pathway | 5 of 110 | 0.77 | 0.0281 |
| 21 | [cfa04530](https://www.kegg.jp/kegg-bin/show_pathway?cfa04530) | Tight junction | 6 of 136 | 0.76 | 0.0177 |
| 22 | [cfa00190](https://www.kegg.jp/kegg-bin/show_pathway?cfa00190) | Oxidative phosphorylation | 5 of 117 | 0.74 | 0.0317 |
| 23 | [cfa05014](https://www.kegg.jp/kegg-bin/show_pathway?cfa05014) | Amyotrophic lateral sclerosis | 13 of 316 | 0.73 | 8.80e^-05^ |
| 24 | [cfa05016](https://www.kegg.jp/kegg-bin/show_pathway?cfa05016) | Huntington disease | 11 of 271 | 0.72 | 0.00056 |
| 25 | [cfa04144](https://www.kegg.jp/kegg-bin/show_pathway?cfa04144) | Endocytosis | 7 of 205 | 0.65 | 0.0245 |
| 26 | [cfa04015](https://www.kegg.jp/kegg-bin/show_pathway?cfa04015) | Rap1 signaling pathway | 6 of 183 | 0.63 | 0.039 |
| 27 | [cfa05012](https://www.kegg.jp/kegg-bin/show_pathway?cfa05012) | Parkinson disease | 7 of 215 | 0.62 | 0.0261 |
| 28 | [cfa05010](https://www.kegg.jp/kegg-bin/show_pathway?cfa05010) | Alzheimer disease | 10 of 322 | 0.6 | 0.0069 |
| 29 | [cfa05020](https://www.kegg.jp/kegg-bin/show_pathway?cfa05020) | Prion disease | 7 of 230 | 0.6 | 0.0317 |
| 30 | [cfa01100](https://www.kegg.jp/kegg-bin/show_pathway?cfa01100) | Metabolic pathways | 30 of 1220 | 0.5 | 3.39e^-06^ |
