# Supplementary material for: Proteomic and phosphoproteomic analysis of rabies pathogenesis in the clinical canine brain and identification of a kinase inhibitor as a potential repurposed antiviral agent
Source: PLoS One. 2025 Jun 27;20(6):e0323931. doi: 10.1371/journal.pone.0323931 (PMC12204518; doi:10.1371/journal.pone.0323931)

Original images for blots

Detection of protein phosphorylation by western blot (S2 Fig.)

Neuro-2a cells were cultured and treated with 5 kinase inhibitor drugs for 0, 16, 24, 48, and 72 h. Protein was extracted from treated cells and 30 µg of protein was subjected to western blot analysis using anti-phosphoserine antibody as the primary antibody. The chemiluminescence substrate (Super Signal West Pico PLUS) was used to detect immunoreactive bands with chemiluminescence module of ChemiDoc imaging system.

1. 7.5 µM Sunitinib malate (S2 Fig. A) - Exposure time 6 min.

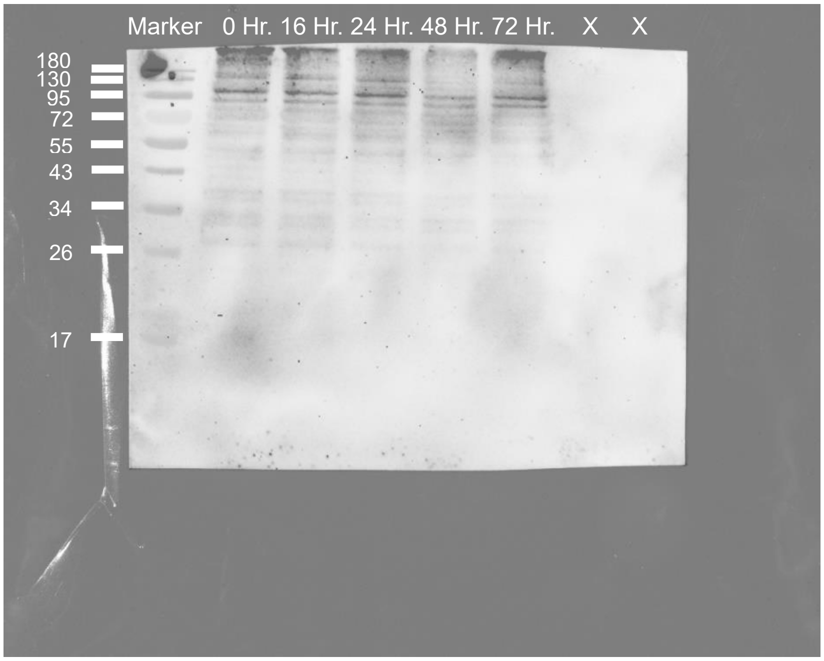

2. 7.5 µM Silmitasertib (S2 Fig. B) - Exposure time 10 min.

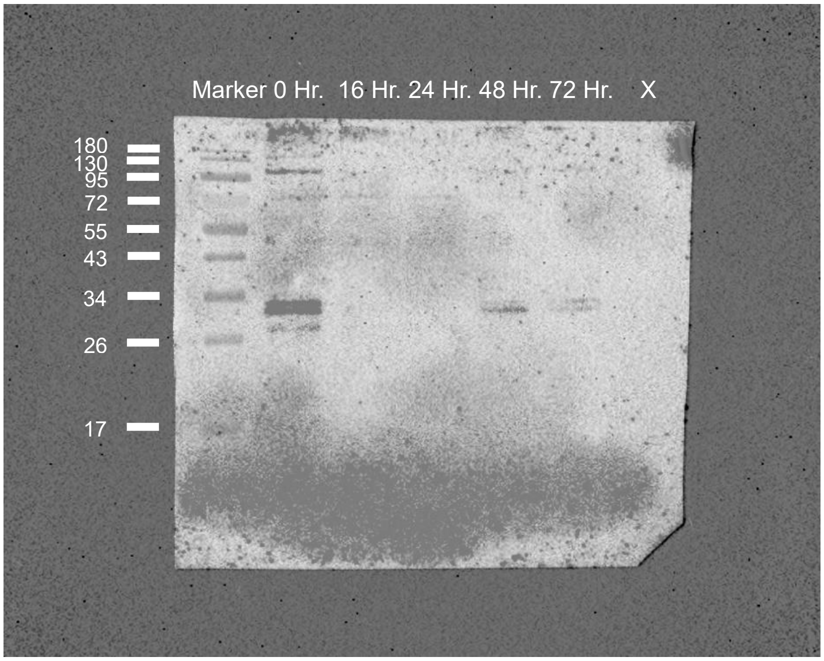

3. 7.5 µM Chelerythrine (S2 Fig. C) - Exposure time 10 min.

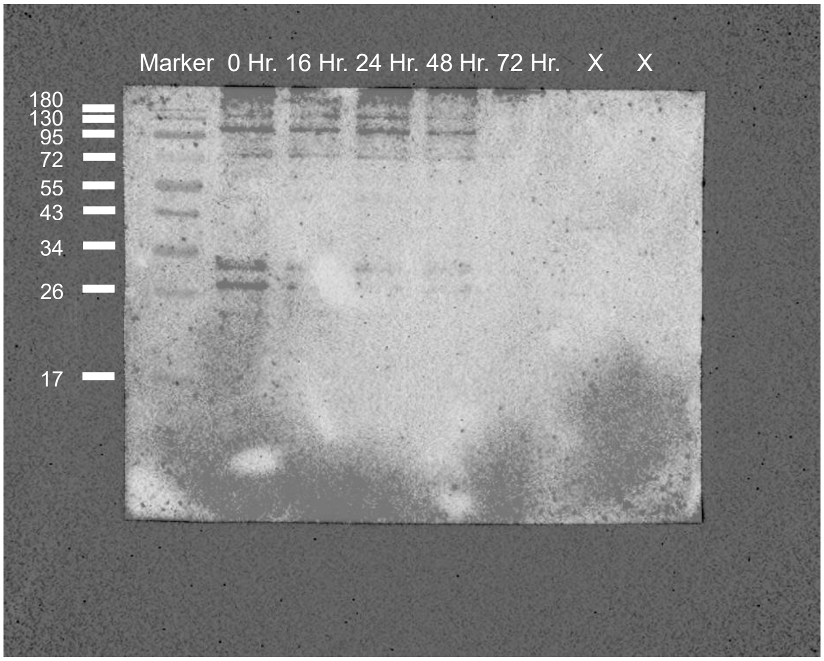

4. 7.5 µM Rottlerin (S2 Fig. D) - Exposure time 6 min.

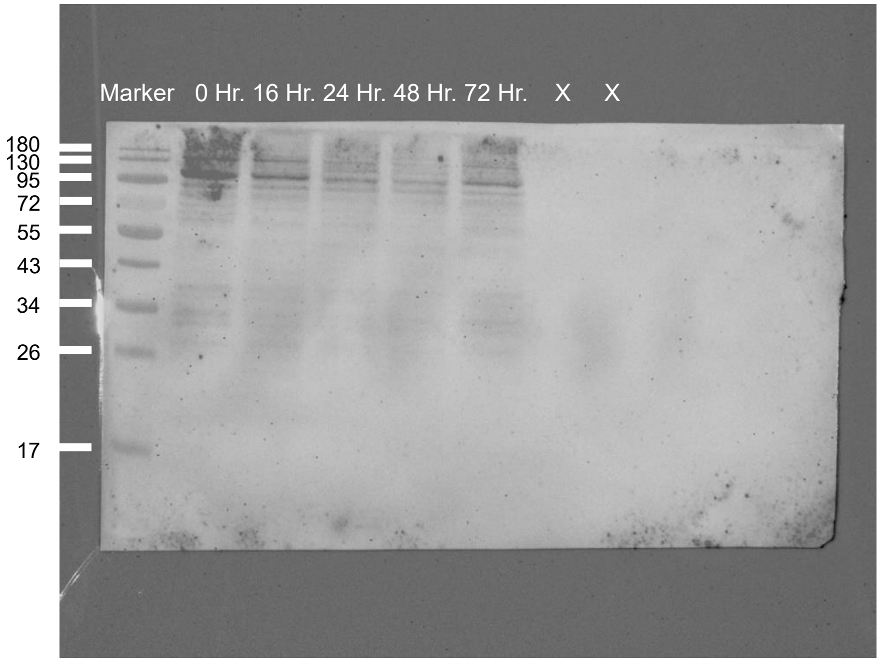

5. 10 µM DMAT (S2 Fig. E) - Exposure time 6 min.

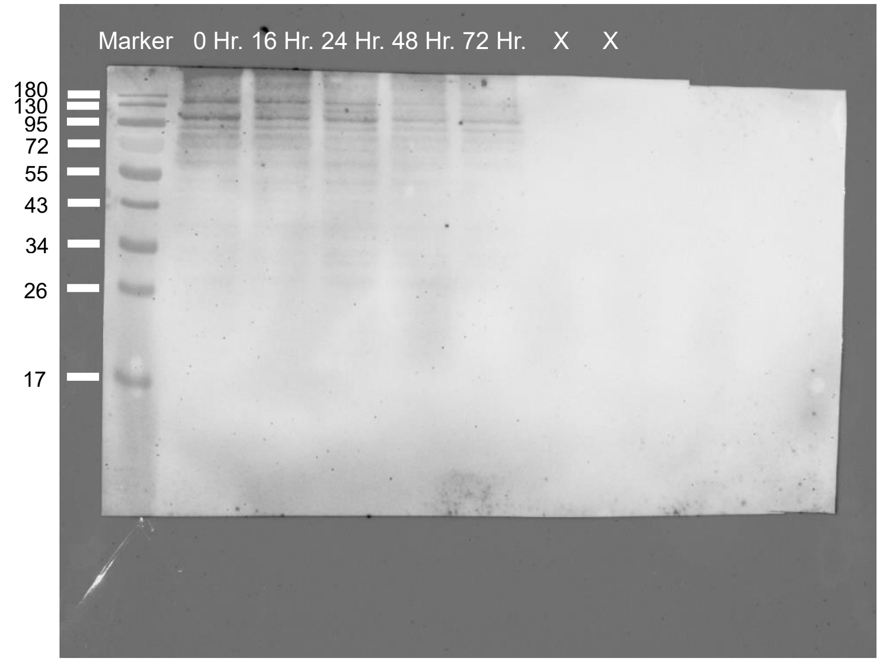

Supplement: S1 File — (PDF) [file pone.0323931.s007.pdf]
